# Supplementary material for: Chaperonin recognition of protein dynamics drives drug resistance
Source: bioRxiv. 2026 Jun 7:2026.06.03.729952. Preprint. [Version 1] doi: 10.64898/2026.06.03.729952 (PMC13251950; doi:10.64898/2026.06.03.729952)
Supplement: Supplement 1 [file media-1.pdf]

Supplementary Materials for

Chaperonin recognition of protein dynamics drives drug resistance

Junlang Liu[1], Zhihui Qi[1], João V. Rodrigues[1], Zhouyu Zhang[1], Qianxiang Xu[1], Abhinav Dubey[2,3], Harrison K. Wang[4,5], Dongtao Cui[1], Zeyang Li[1], Fatima Aly[1], Doeke R. Hekstra[4,6], Eugene I. Shakhnovich[1]*

[1]Department of Chemistry and Chemical Biology, Harvard University, Cambridge, MA, USA. [2]Department of Cancer Biology, Dana-Farber Cancer Institute, Boston, MA, USA. [3]Department of Biological Chemistry and Molecular Pharmacology, Harvard Medical School, Boston, MA, USA. [4]Department of Molecular and Cellular Biology, Harvard University, Cambridge, MA, USA. [5]Graduate Program in Biophysics, Harvard University, Boston, MA, USA. [6]School of Engineering and Applied Sciences, Harvard University, Boston, MA, USA.

* Corresponding author. E-mail: shakhnovich@chemistry.harvard.edu

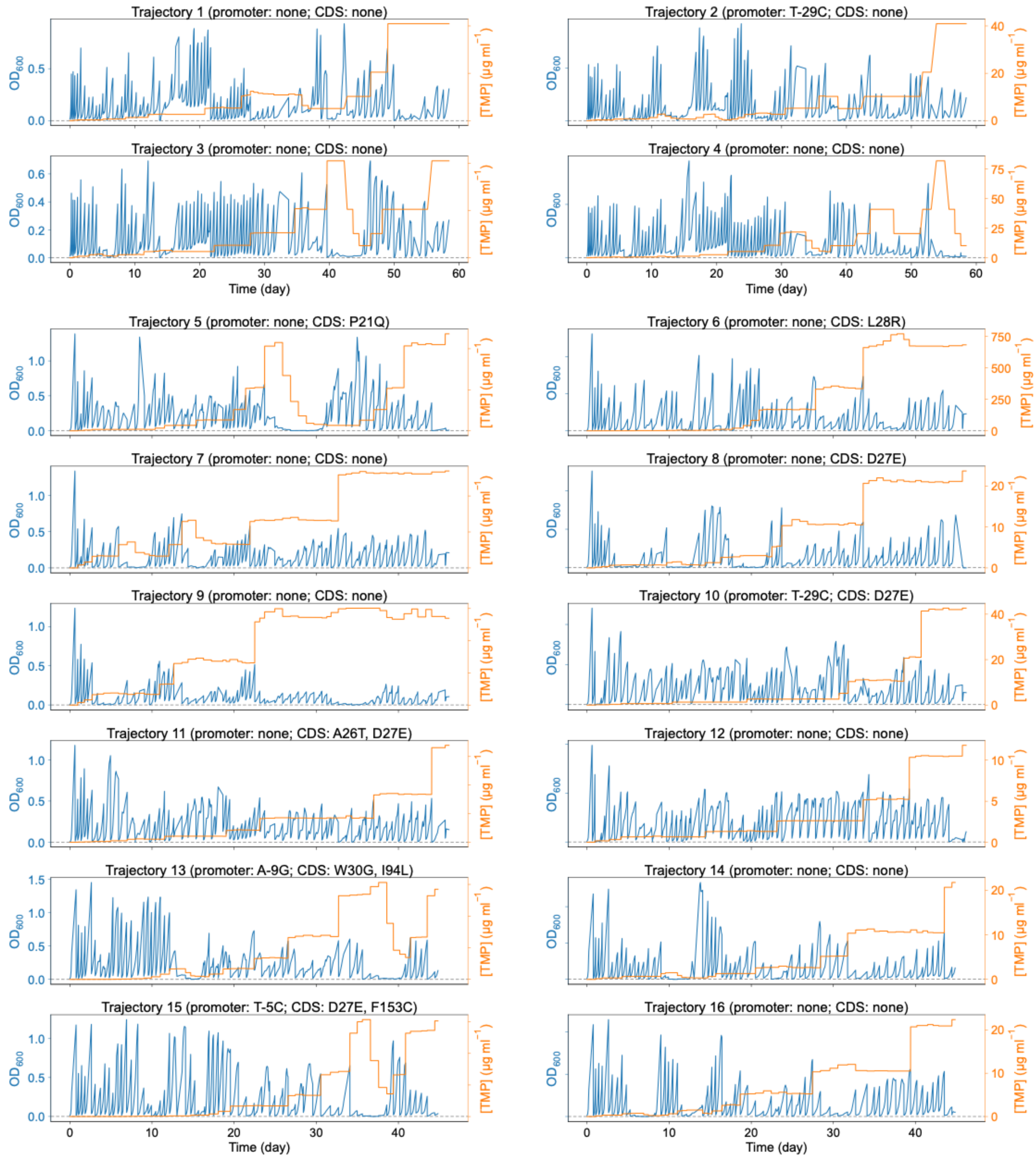

Supplementary Fig. 1. Trajectories of TMP concentration and cell density for control group during evolution. Sixteen parallel evolutionary trajectories were maintained for 45 days (~60 passages), with trajectories 1–4 extended to 60 days (~80 passages). Genomic mutations were identified from strains isolated at passage 60 for all trajectories. Trajectories 1–4 underwent additional sequencing at passage 80; any novel mutations emerging at this extended time point are explicitly annotated, whereas the absence of such labels indicates no further genetic changes were detected.

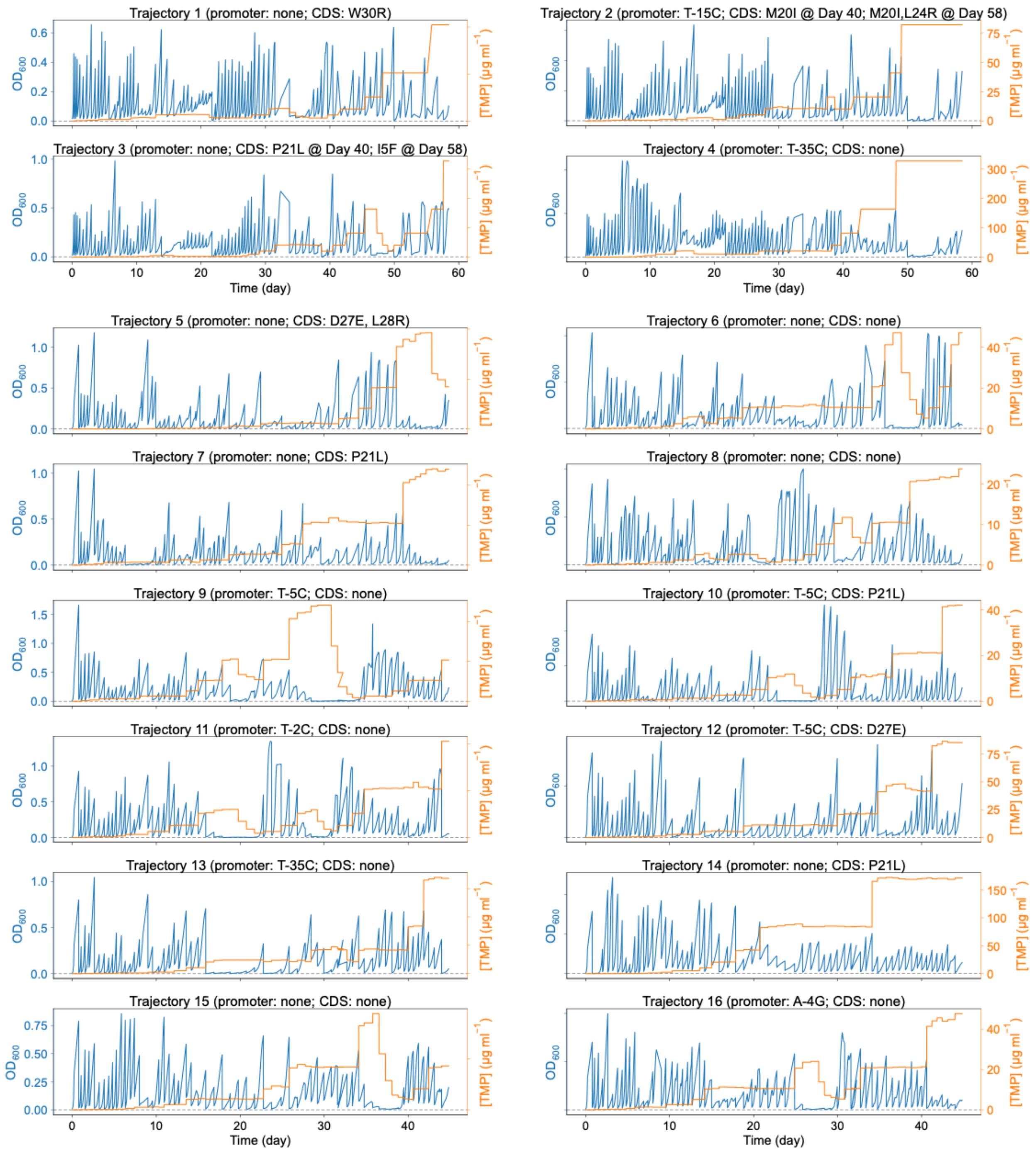

Supplementary Fig. 2. Trajectories of TMP concentration and cell density for GroEL/S overexpression group during evolution. Sixteen parallel evolutionary trajectories were maintained for 45 days (~60 passages), with trajectories 1–4 extended to 60 days (~80 passages). Genomic mutations were identified from strains isolated at passage 60 for all trajectories. Trajectories 1–4 underwent additional sequencing at passage 80; any novel mutations emerging at this extended time point are explicitly annotated, whereas the absence of such labels indicates no further genetic changes were detected.

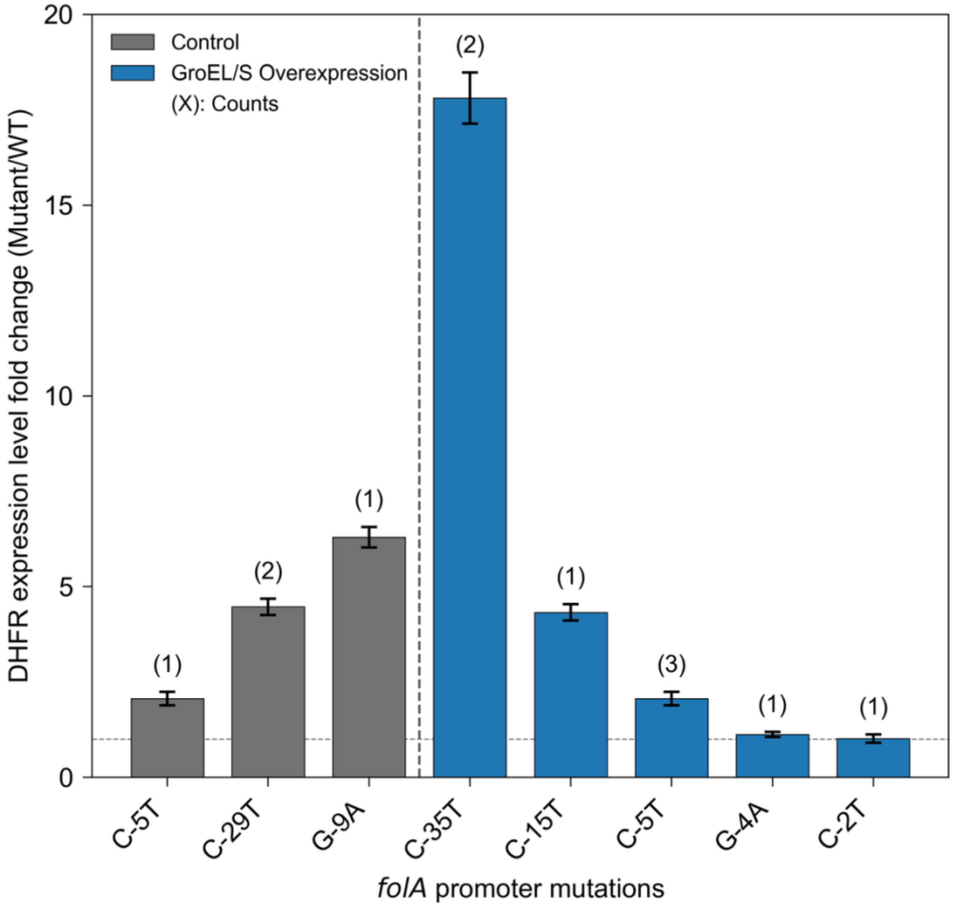

Supplementary Fig. 3. Fold change in DHFR expression driven by *folA* promoter mutations identified through laboratory directed evolution. GFP fluorescence was measured as a proxy for DHFR expression in *E. coli* DH5α strains harboring either the wild-type pfolA-GFP plasmid or its mutant variants. Briefly, overnight cultures were inoculated from frozen stocks into LB medium supplemented with 50 μg ml^{-1} kanamycin and incubated for 12–16 h at 37 °C and 250 rpm. Starter cultures were subsequently diluted 1:100 (v/v) into fresh LB medium with with 50 μg ml^{-1} kanamycin and grown for 6 h under identical conditions. Cell suspensions were normalized to an OD_{600} of 0.1 prior to fluorescence measurements. Background-subtracted fluorescence intensities for each mutant were normalized to the wild-type promoter to calculate the fold change in expression. Data represent the mean ± SEM (standard error of the mean) of triplicate. Counts indicate the number of independent trajectories out of 16 total per group that acquired the specified mutations.

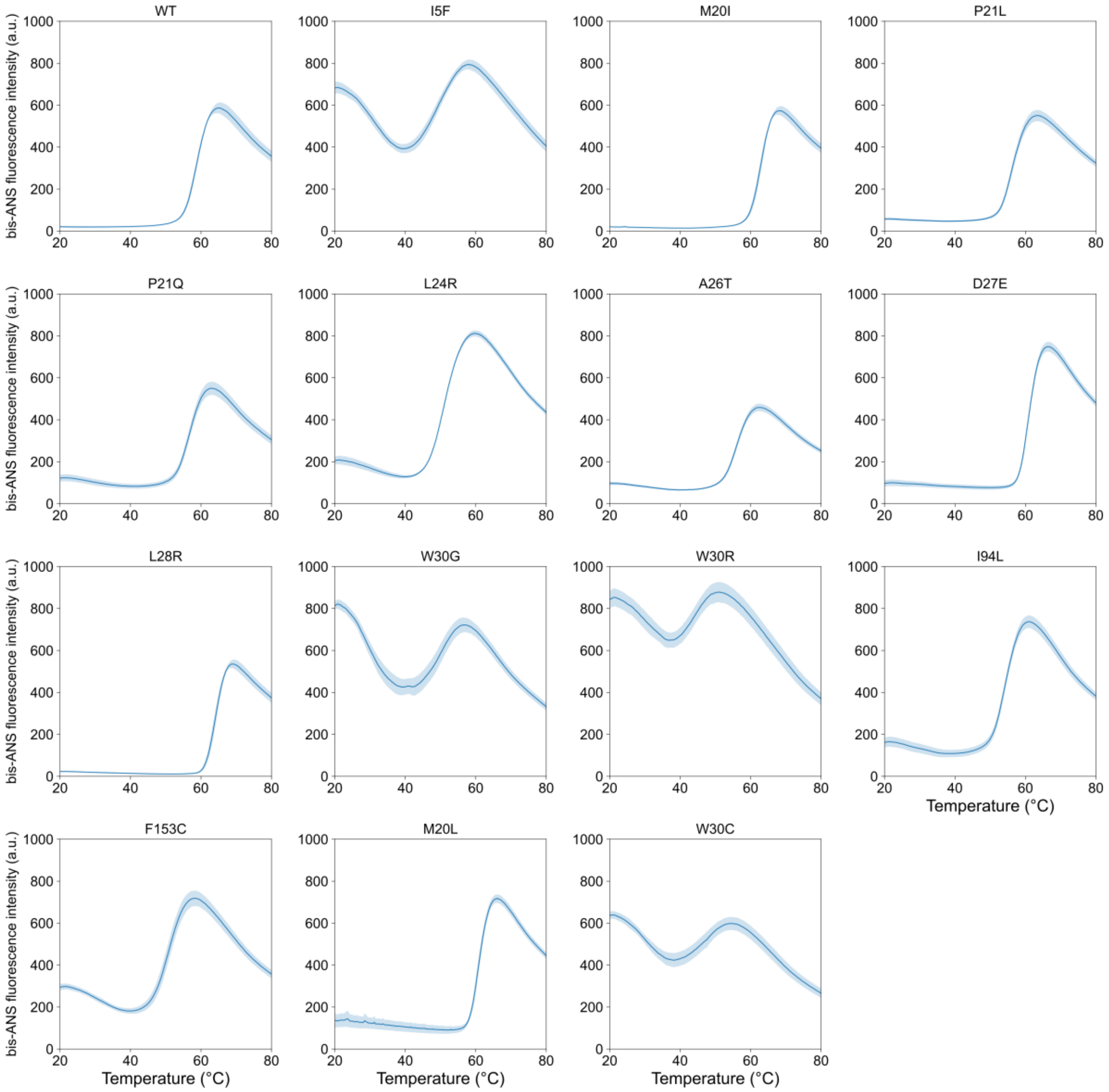

Supplementary Fig. 4. Thermal unfolding profiles of WT DHFR and mutants monitored by bis-ANS fluorescence. Solid blue lines represent the mean fluorescence intensity (arbitrary units, a.u.) across 3 independent replicates for each DHFR. The light blue shaded regions indicate the ± SEM.

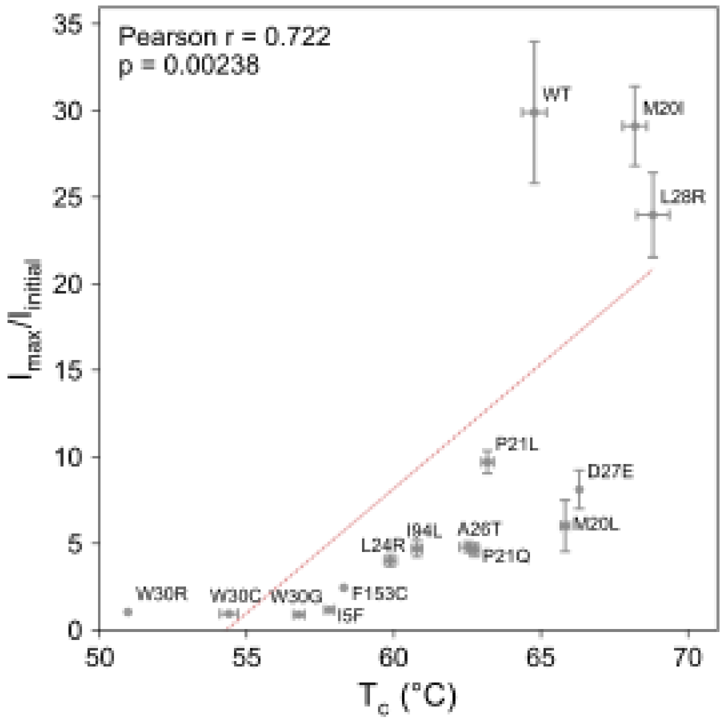

Supplementary Fig. 5. Correlation between critical temperature (T_c) and molten globule degree ($I_{max}/I_{initial}$) across DHFR variants. $I_{max}/I_{initial}$ and T_c values were taken from Fig. 2b, revealing a statistically significant positive correlation (Pearson $r = 0.722$, $p = 0.00238$). Mutants with smaller $I_{max}/I_{initial}$ values exhibit more pronounced molten globule state, accompanied by a correspondingly lower T_c. The dashed line is shown as a guide to the eye.

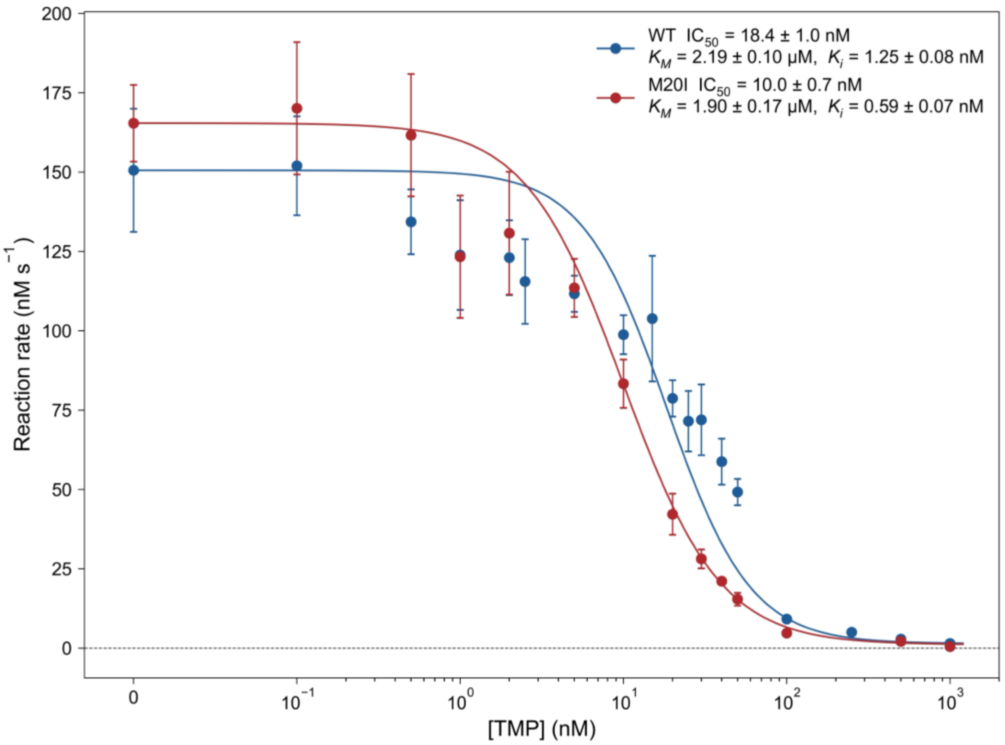

Supplementary Fig. 6. TMP dose-response curves for WT and M20I DHFR. *In vitro* reaction rates for WT and M20I DHFR were measured as a function of TMP concentration. Data represent the mean ± SEM of four independent replicates. Solid lines represent fits used to determine IC_{50} and K_i values.

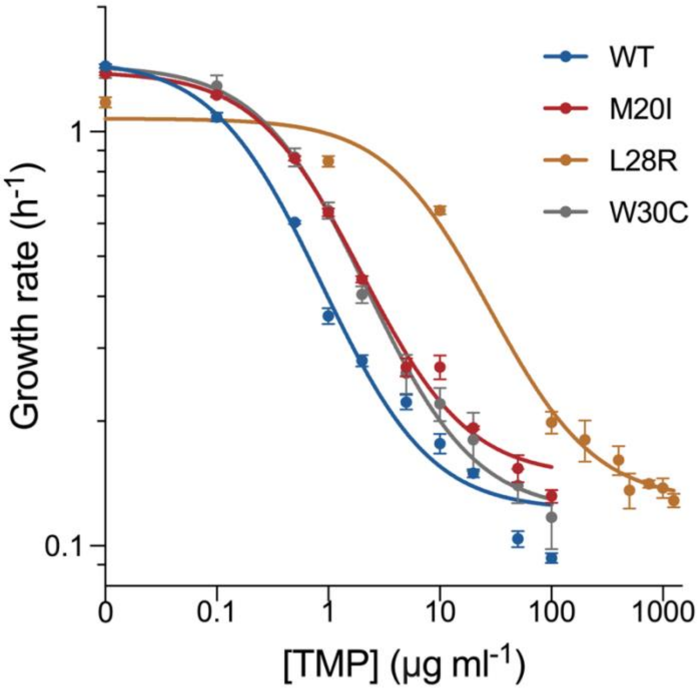

Supplementary Fig. 7. TMP-response curves of WT and mutant DHFR strains. Exponential growth rates (h^{-1}) measured at 37 °C are plotted as a function of TMP concentration (μg ml^{-1}) for WT, M20I, L28R, and W30C variants. Data points and error bars represent the mean ± SEM of 6 replicates. Solid lines indicate three-parameter logistic dose-response fits as described in Methods section.

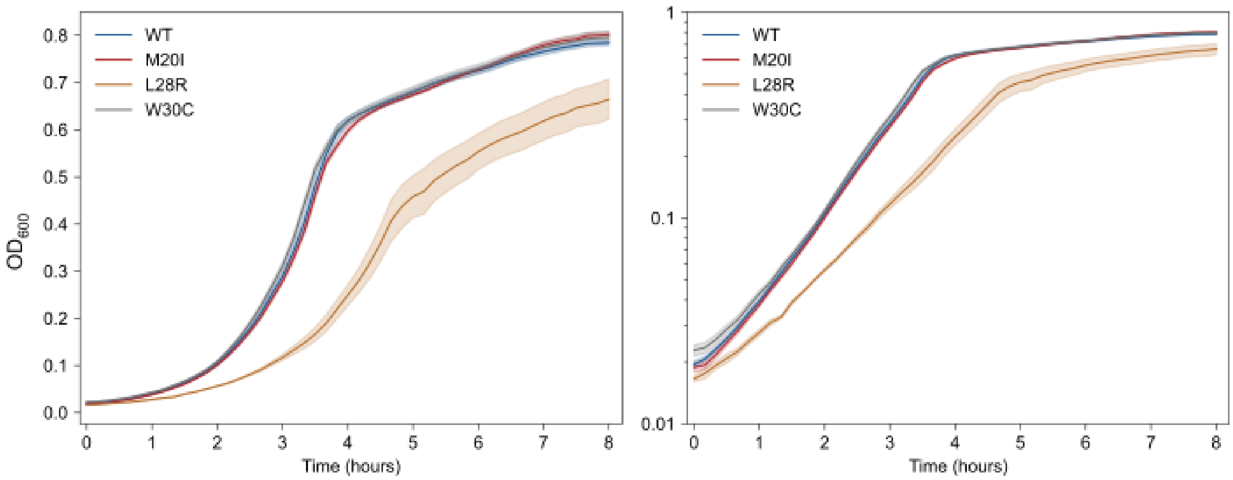

Supplementary Fig. 8. Growth curves of WT and mutant DHFR strains. Time-course OD_{600} measurements of *E. coli* BW25113 strains expressing WT, M20I, L28R or W30C DHFR chromosomally in the absence of antibiotic stress at 37 °C. The left panel shows OD_{600} on a linear scale, and the right panel shows the same data on a semi-log scale to highlight the exponential growth phase. Solid lines and shaded regions represent the mean ± SEM of 6 replicates.

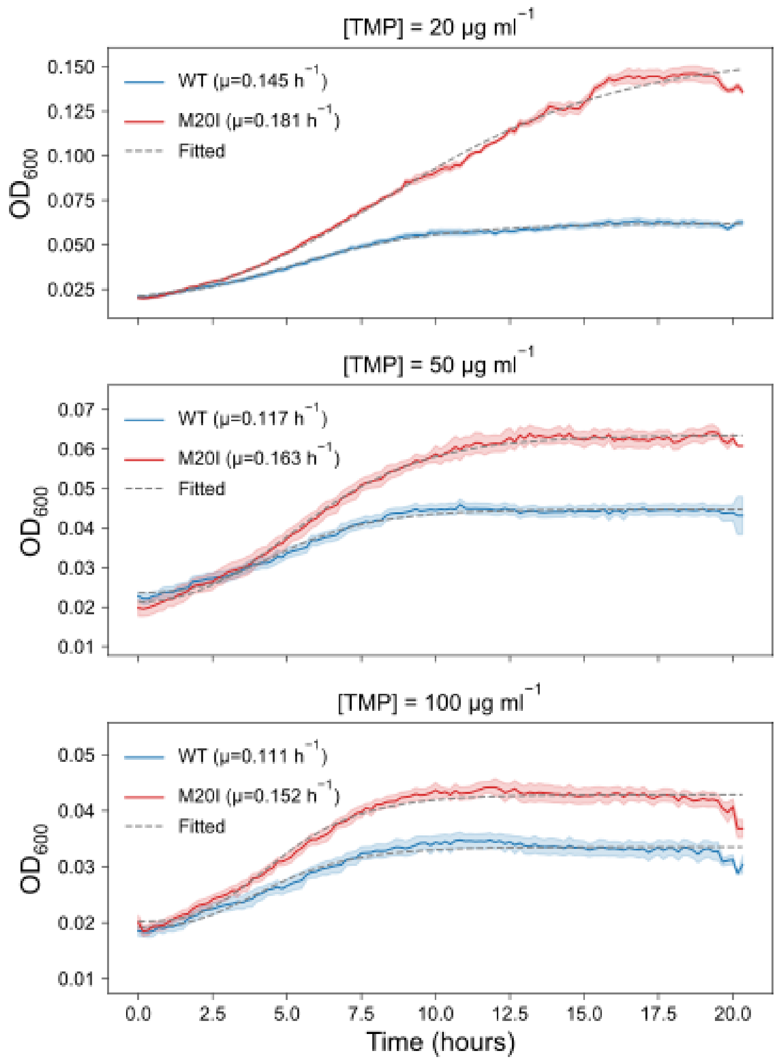

Supplementary Fig. 9. TMP concentration-dependent growth curves for WT and M20I DHFR. Cell density (OD_{600}) of WT and M20I strains was measured over 20 h in the presence of 20, 50, and 100 μg ml^{-1} TMP at 37 °C. Solid lines and shaded regions represent the mean ± SEM of 6 replicates. Dashed lines represent the four-parameter Gompertz growth model fit to the experimental data, as detailed in Methods section. μ is maximum specific growth rate obtained by fitting the mean growth curves.

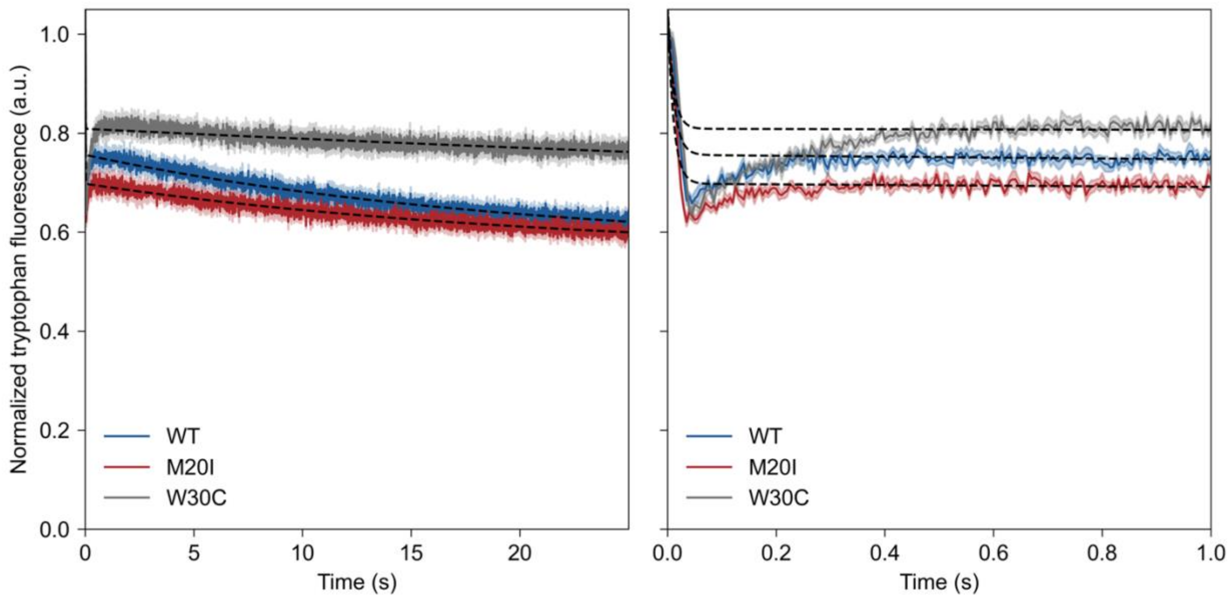

Supplementary Fig. 10. Folding kinetics of DHFR variants monitored by stopped-flow tryptophan fluorescence. The right panel displays a magnified view of the initial one-second folding phase from the full trace shown on the left. Solid lines and shaded regions represent the mean ± SEM of 7 independent replicates. Dashed black lines indicate double-exponential kinetic fits, as detailed in the Methods section.

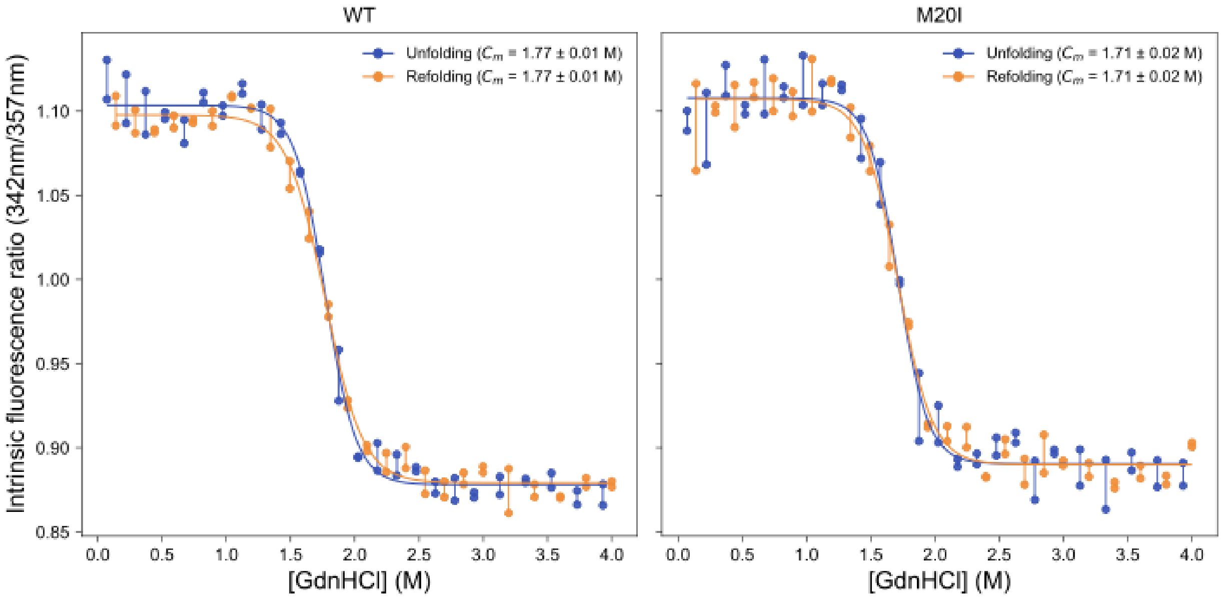

Supplementary Fig. 11. Equilibrium unfolding and refolding of WT and M20I DHFR. GdnHCl-induced denaturation of WT and M20I DHFR monitored by intrinsic fluorescence. Overlapping forward and reverse curves demonstrate thermodynamic reversibility. Calculated transition midpoints (C_m) are inset. Data points represent individual measurements from two replicates at each GdnHCl concentration and are connected by lines to guide the eye. Solid lines indicate the two-state model fit as described in Methods section.

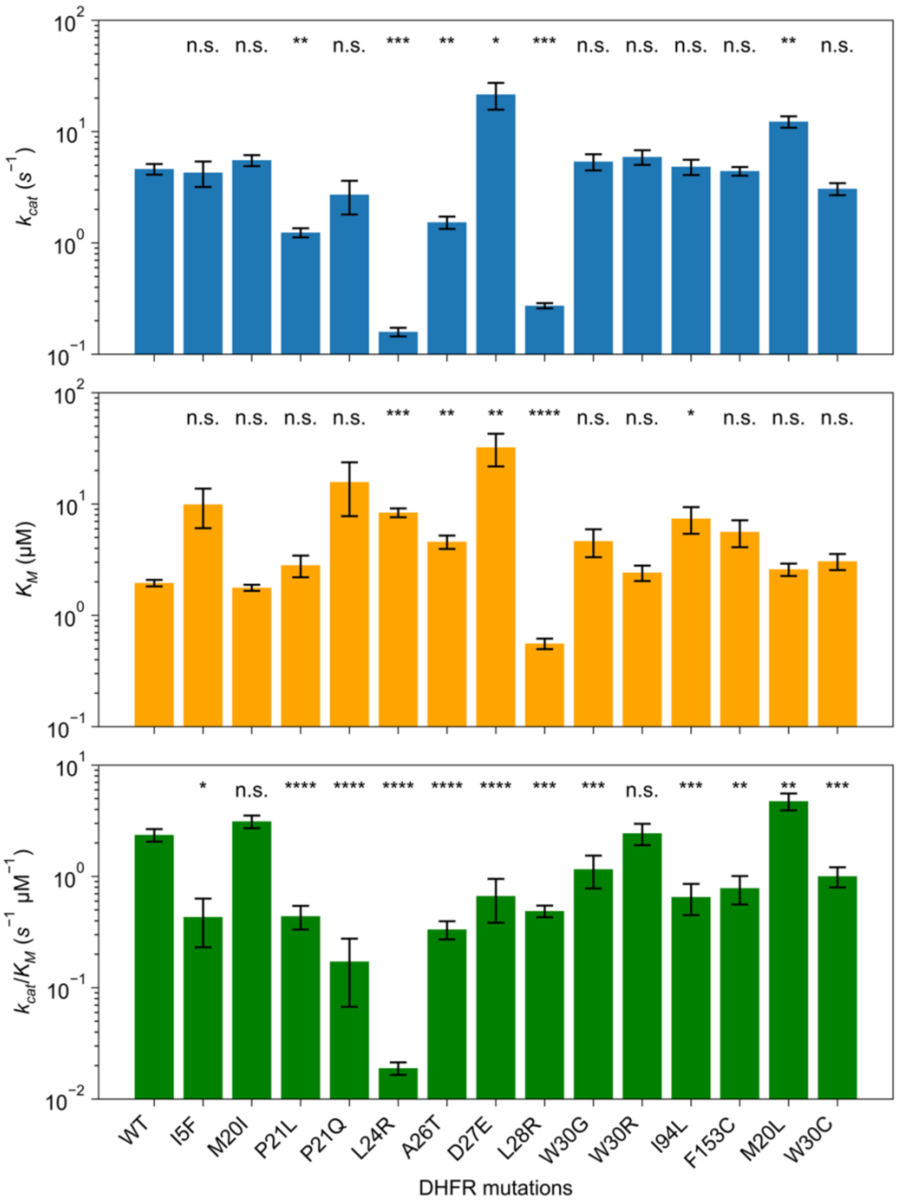

Supplementary Fig. 12. Kinetic parameters (k_{cat}, K_M, and k_{cat}/K_M) for all DHFR variants. Statistical significance was determined by comparing each individual mutant to WT.

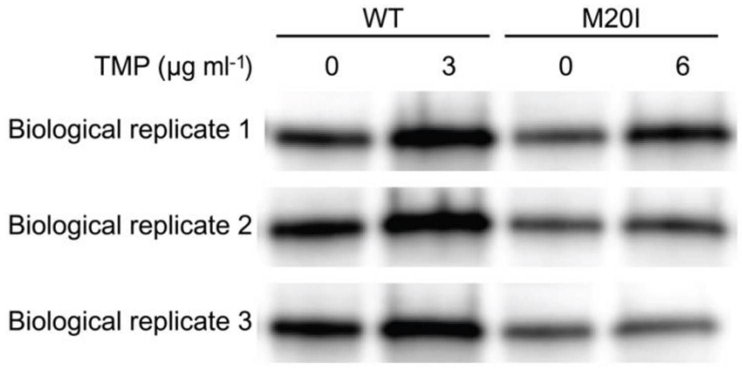

Supplementary Fig. 13. Expression levels of chromosomally encoded WT and M20I DHFR. Western blot analysis of DHFR from *E. coli* BW25113 expressing either WT or M20I DHFR chromosomally. Cultures were grown in the absence of TMP (0 µg ml^{-1}) or at a TMP concentration corresponding to the respective IC_{50} for each strain (3 µg ml^{-1} for WT and 6 µg ml^{-1} for M20I). Data represent three independent biological replicates.

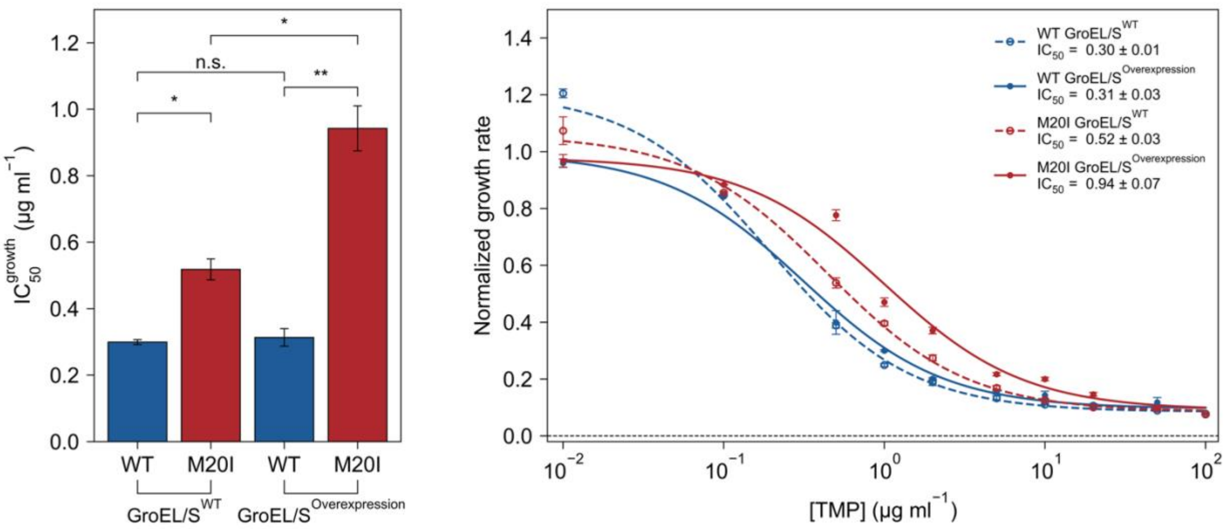

Supplementary Fig. 14. GroEL/S overexpression selectively enhances TMP resistance of M20I. (Left) IC_{50} values derived from cell growth assays for WT and M20I DHFR strains in endogenous (GroEL/S^{WT}) versus GroEL/S overexpression backgrounds measured at 37 °C. Bars denote the mean ± SEM of 3 biological replicates. Statistical analysis highlights the specific enhancement of M20I resistance upon GroEL/S overexpression. **(Right)** Corresponding TMP dose-response curves at 37 °C. Normalized growth rates of WT and M20I strains are plotted as a function of TMP concentration (μg ml^{-1}). Dashed and solid lines represent three-parameter logistic fits for strains in the endogenous (GroEL/S^{WT}) and GroEL/S overexpression backgrounds, respectively. These fits were used to derive the inset IC_{50} values, as detailed in the Methods section. Data points and error bars represent the mean ± SEM of 3 biological replicates.

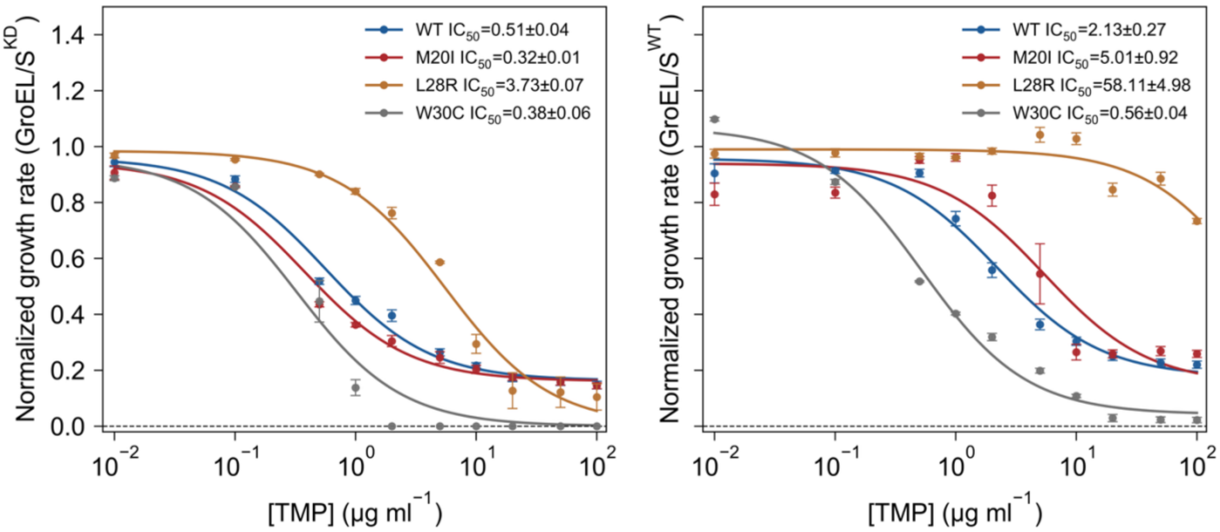

Supplementary Fig. 15. TMP-response curves of DHFR variants under GroEL/S knockdown and endogenous conditions. Normalized growth rates of WT, M20I, L28R, and W30C strains measured at 37 °C are plotted as a function of TMP concentration (μg ml^{-1}) in GroEL/S knockdown (left, GroEL/S^{KD}) and endogenous (right, GroEL/S^{WT}) backgrounds. Solid lines indicate three-parameter logistic dose-response fits used to derive the inset IC_{50} values, as detailed in Methods section. Data points and error bars represent the mean ± SEM of 6 replicates.

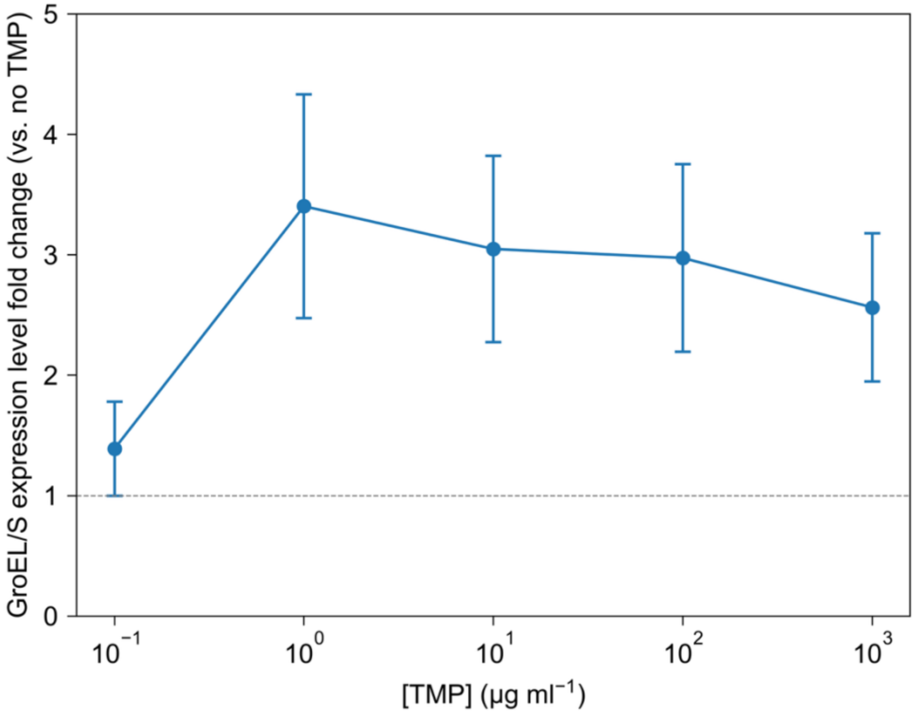

Supplementary Fig. 16. Fold change in GroEL/S expression under varying concentrations of TMP. GFP fluorescence was measured as a proxy for GroEL/S expression in *E. coli* DH5α strains harboring the pgroS-GFP plasmid. Briefly, overnight cultures were inoculated from frozen stocks into LB medium supplemented with 50 μg ml^{-1} kanamycin and incubated for 12–16 h at 37 °C and 250 rpm. Starter cultures were subsequently diluted 1:100 (v/v) into fresh LB medium containing 50 μg ml^{-1} kanamycin and the respective TMP concentrations, followed by growth for 6 h at 37 °C and 250 rpm to allow for cellular adaptation. Cell suspensions were normalized to an OD_{600} of 0.1 prior to fluorescence measurements. To calculate the fold change in expression, background-subtracted fluorescence intensities for each TMP concentration were normalized to those measured in the untreated control (LB medium without TMP). Data represent the mean ± SEM of triplicate.

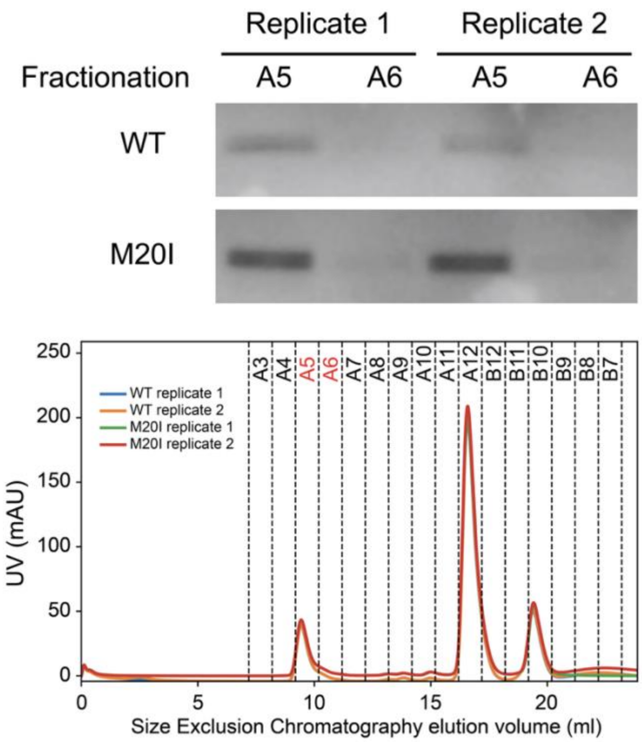

Supplementary Fig. 17. SEC co-elution of GroEL and DHFR ternary complexes. SEC chromatograms (bottom) of GroEL–DHFR mixtures capture the high-molecular-weight GroEL peak (red fractions A5, A6), unbound DHFR (second peak), and small-molecule ligands (third peak). Western blotting of the A5 and A6 fractions (top) reveals that while both WT and M20I DHFR co-elute with GroEL, the M20I variant exhibits a darker band intensity, indicating an enhanced physical interaction. All assays were performed in independent duplicates.

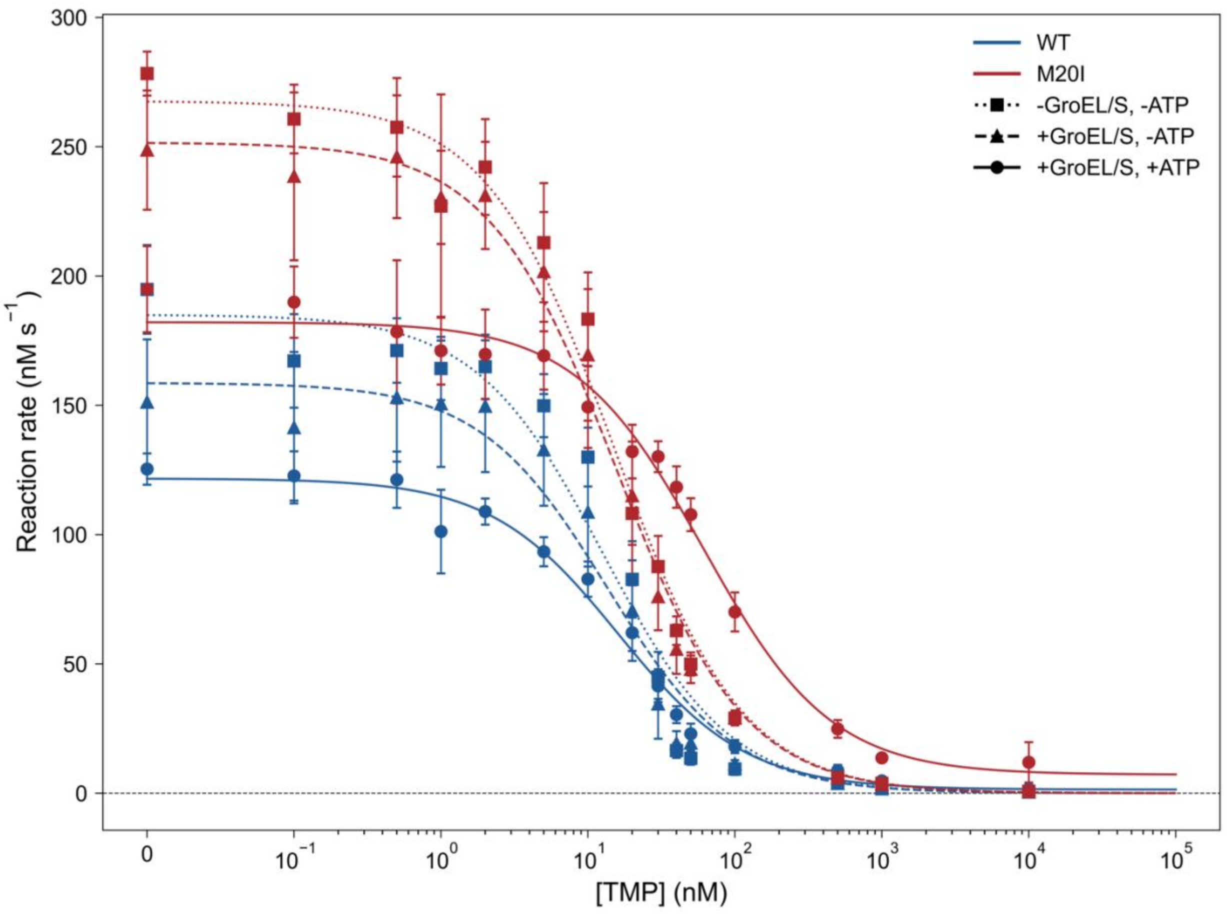

Supplementary Fig. 18. GroEL/S-mediated rescue of DHFR activity. *In vitro* reaction rates of WT and M20I DHFR plotted as a function of TMP concentration. Reactions were evaluated without chaperone (-GroEL/S, -ATP), with inactive chaperone (+GroEL/S, -ATP), and under active refolding conditions (+GroEL/S, +ATP). The M20I variant exhibits substantial functional recovery exclusively under active rescue conditions, even under extremely high TMP concentrations. Data represent the mean ± SEM of three independent replicates.

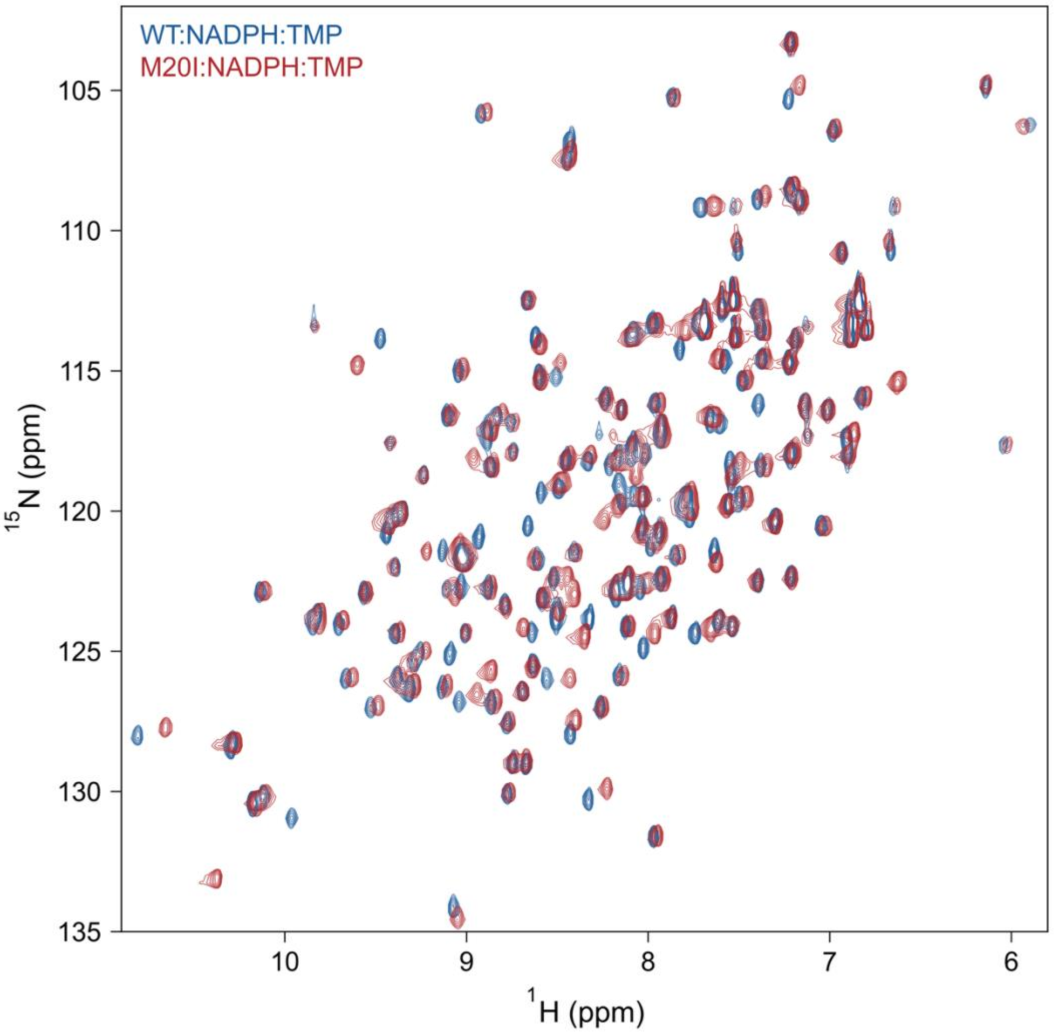

Supplementary Fig. 19. 2D ^{1}H-^{15}N HSQC spectra of WT and M20I DHFR. Spectral overlay of the WT (blue) and M20I (red) DHFR ternary complexes fully liganded with NADPH and TMP. Data were acquired at 298 K at 600 MHz. The dispersion and alignment of the cross-peaks confirm that the mutant retains the WT global fold while exhibiting specific chemical shift perturbations.

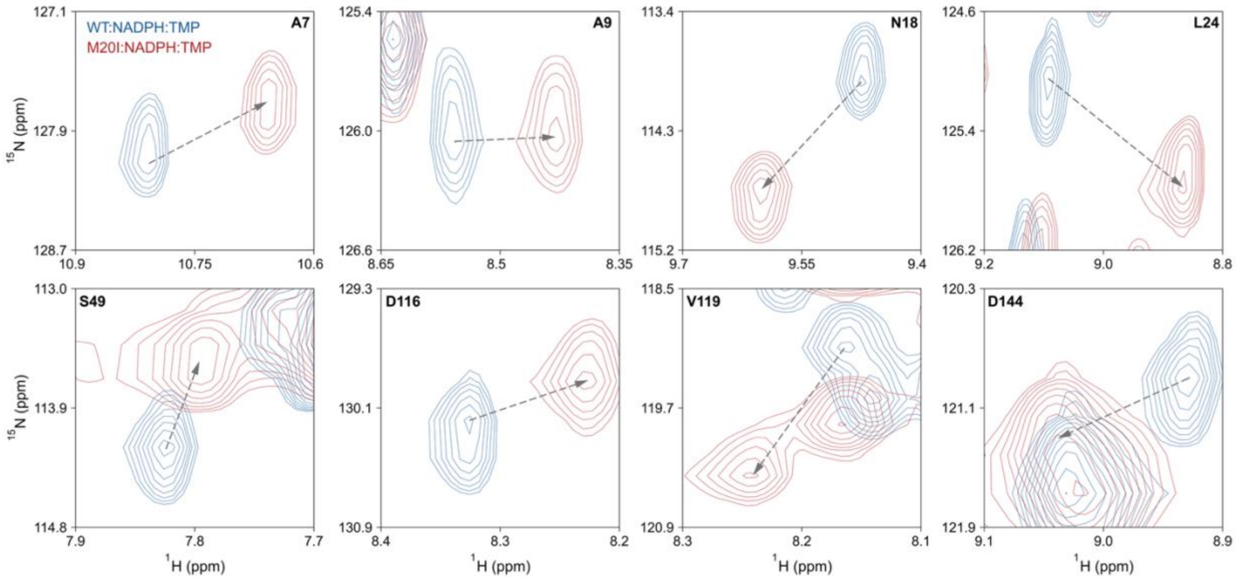

Supplementary Fig. 20. Localized chemical shift perturbations induced by the M20I mutation in DHFR. Expanded regions of the 2D ^{1}H-^{15}N HSQC spectra comparing the WT (blue) and M20I (red) DHFR ternary complexes. Dashed arrows track the movement of selected cross-peaks that exhibit significant weighted chemical shift distances (> 0.2 ppm). These highly perturbed resonances map to critical functional domains, including the M20 loop (A7, A9, N18, L24), the substrate-binding pocket (S49), the F-G loop (D116, V119), and the G-H loop (D144).

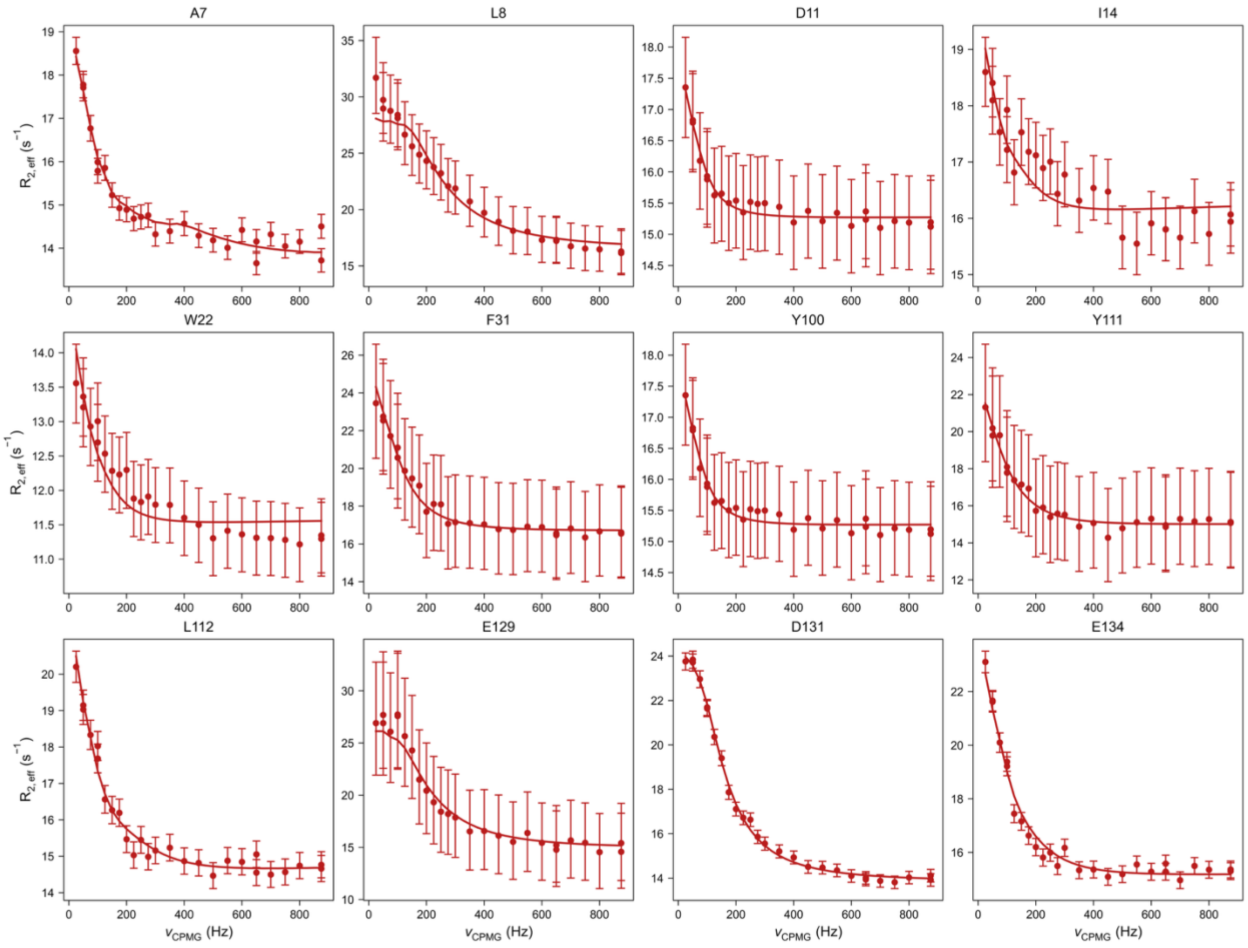

Supplementary Fig. 21. ^{15}N CPMG relaxation dispersion curves of M20I residues. Plots of $R_{2,eff}$ vs. ν_{CPMG} for selected backbone amides undergoing millisecond-level chemical exchange. These residues map to the M20 loop (A7, L8, D11, I14, W22), the substrate binding pocket (F31), residues interacting with the M20 loop (Y100, Y111, L112), and the F-G loop (E129, D131, E134). Solid lines denote global fits applied to the dispersion data, driven by residues A7, L112, E129, and D131, to extract overarching exchange parameters. Error bars reflect uncertainties derived from PeakFit and ChemEx analysis. The reduced χ^2 for the global fit is 0.48.

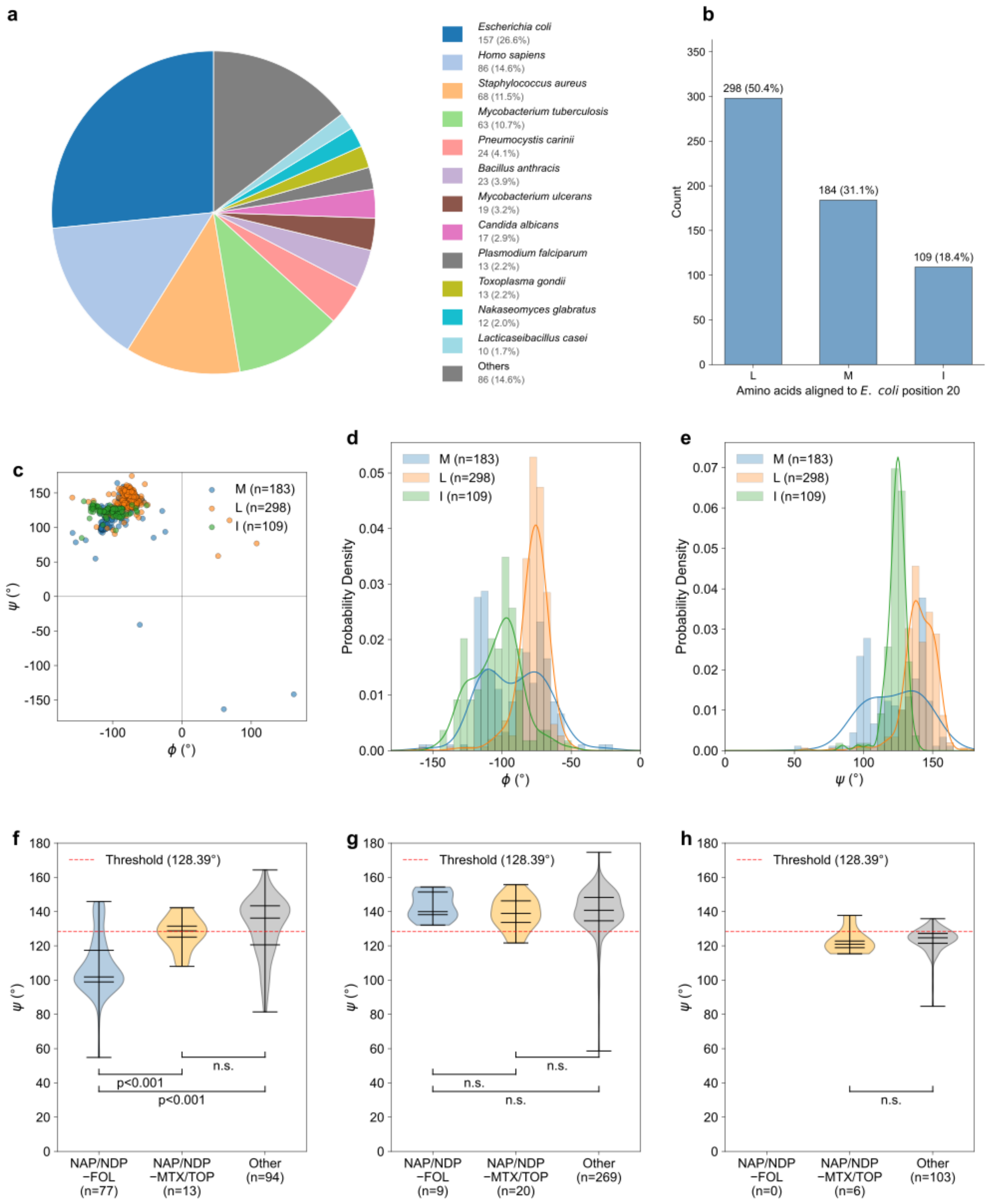

Supplementary Fig. 22. Meta-analysis of DHFR position 20 conformational plasticity across the PDB. Analyses utilize all available DHFR structures (n = 591) with sequences aligned to *E. coli* DHFR (UniProt: P0ABQ4). **a, b**, Distribution of source species (**a**) and amino acid composition (**b**) at the aligned position 20. In **a**, species with fewer than 10 available structures are

grouped as “Others”. **c**, Ramachandran plot (ϕ vs. ψ) for structures containing methionine (M), leucine (L), or isoleucine (I) at position 20. **d, e**, Probability density functions for the ϕ (**d**) and ψ (**e**) dihedral angles, highlighting the broad conformational sampling of native M compared to the locally rigid distribution of I. **f–h**, Violin plots detailing the ψ angle distributions for M (**f**), L (**g**), and I (**h**) across distinct ligand environments: NAP/NDP ($NADP^+$/NADPH) paired folate (FOL), methotrexate or trimethoprim (MTX/TOP), or other combinations. The red dashed line (ψ = 128.39°) denotes the boundary between low- and high-ψ M subpopulations, defined by the intersection of a two-Gaussian mixture model fitted to the ψ distribution of M. This reference line illustrates how native M dynamically shifts its conformation to accommodate different ligands, whereas I remains sterically restricted. Inside the violins, black horizontal bars denote the 25th, 50th, and 75th percentiles. *p*-values were determined via two-sided Mann–Whitney U tests (n.s. means not significant).

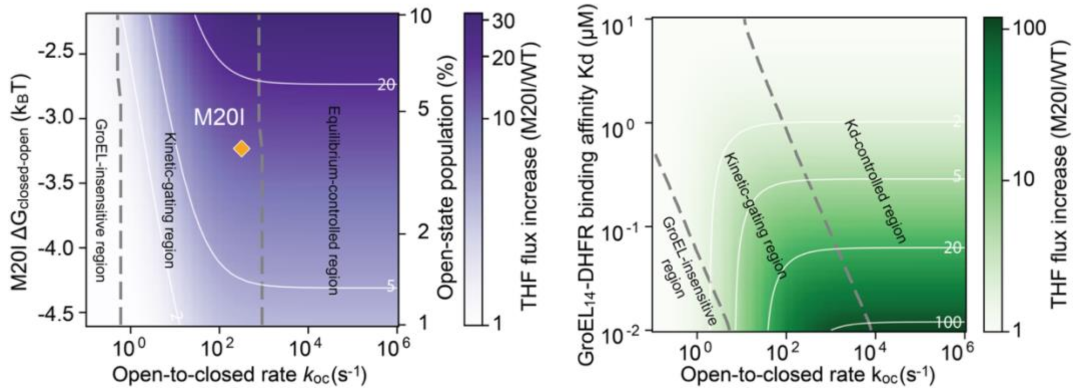

Supplementary Fig. 23. Kinetic modeling of GroEL/S-mediated THF metabolic flux enhancement. (Left) Predicted THF flux increase (M20I/WT) plotted against the open-to-closed transition rate (k_{oc}) and the free-energy difference between the closed and open states of M20I ($\Delta G_{closed\text{-}open}$). The model defines GroEL-insensitive, kinetic-gating, and equilibrium-controlled regimes. The orange diamond denotes the experimental coordinates for M20I, as determined via NMR CPMG measurements. **(Right)** Predicted flux enhancement mapped against k_{oc} and the $GroEL_{14}$–DHFR binding affinity (K_d). This landscape highlights that kinetics predominantly determines the THF flux increase across a wide range of $GroEL_{14}$–DHFR interaction strength.

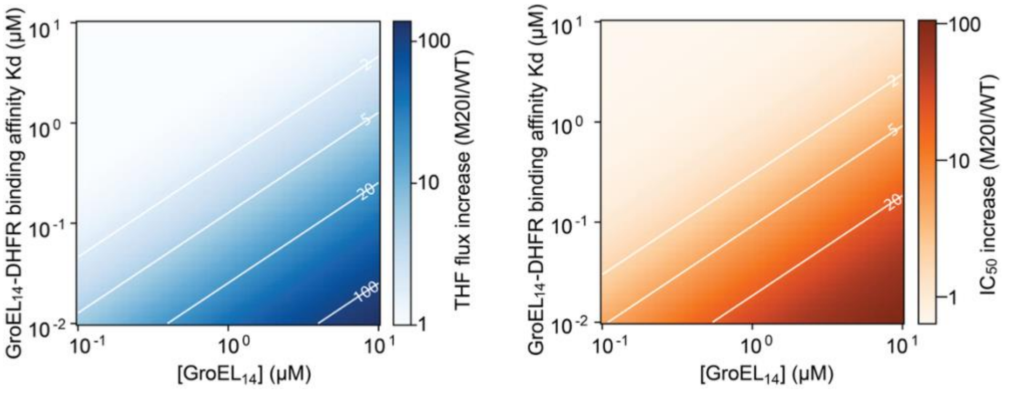

Supplementary Fig. 24. Dependence of M20I functional rescue on $GroEL_{14}$ concentration and binding affinity. (Left) Predicted THF metabolic flux increase of the M20I mutant relative to WT plotted as a function of the $GroEL_{14}$ concentration and the $GroEL_{14}$–DHFR binding affinity (K_d). **(Right)** Predicted IC_{50} increase of M20I relative to WT mapped across the identical K_d and $GroEL_{14}$ concentration parameter space. Both predicted landscapes illustrate that functional and phenotypic rescue scales synergistically with high chaperone availability and strong interaction strength.

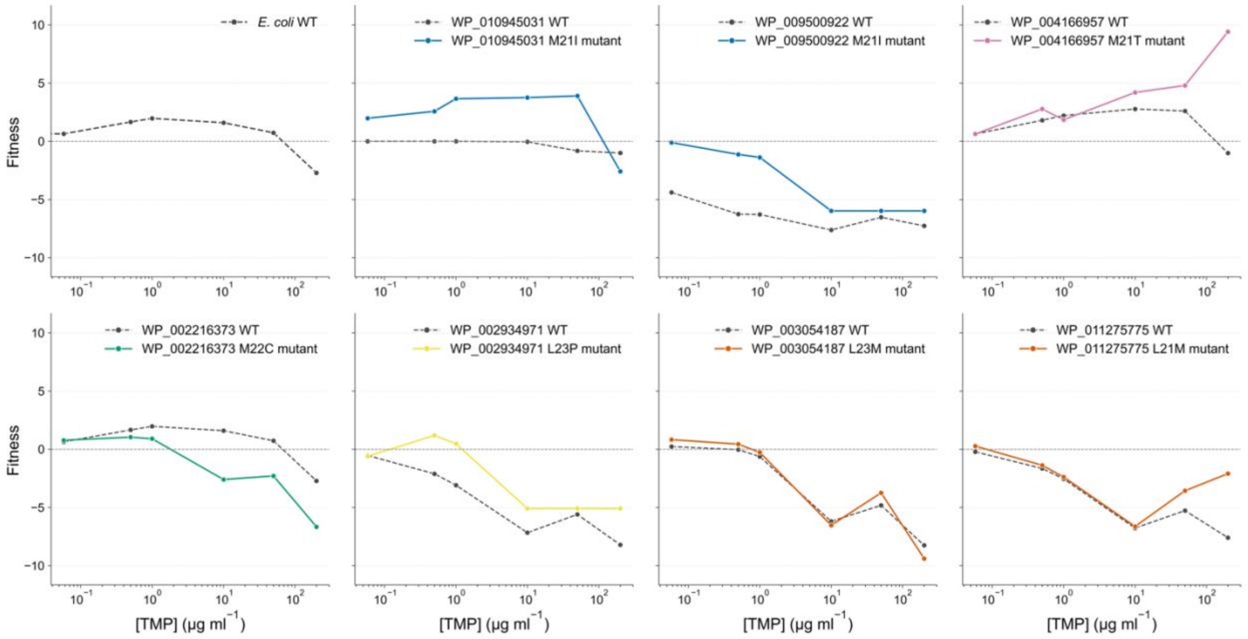

Supplementary Fig. 25. TMP-response curves of all identified position 20 single-substitution mutants. Fitness trajectories across varying TMP concentrations are displayed for all seven single-mutant variants identified in the dataset. Each plotted variant only contains a single amino acid substitution at a site corresponding to reference position 20 in *E. coli* DHFR. Fitness was defined as the dimensionless log2-transformed fold change in normalized barcode counts at each drug concentration relative to the no-drug baseline. Solid colored lines indicate the mutants, while dashed black lines represent their corresponding unmutated wild-type homologs. Notably, across all identified variants, only the M-to-I substitutions exhibit enhanced fitness relative to their wild-type counterparts. The mutation labels provided in each panel (e.g., M21I, L23P) denote the exact amino acid position within that specific homolog's sequence. The *E. coli* WT fitness trajectory is shown in the top-left panel as a baseline reference.

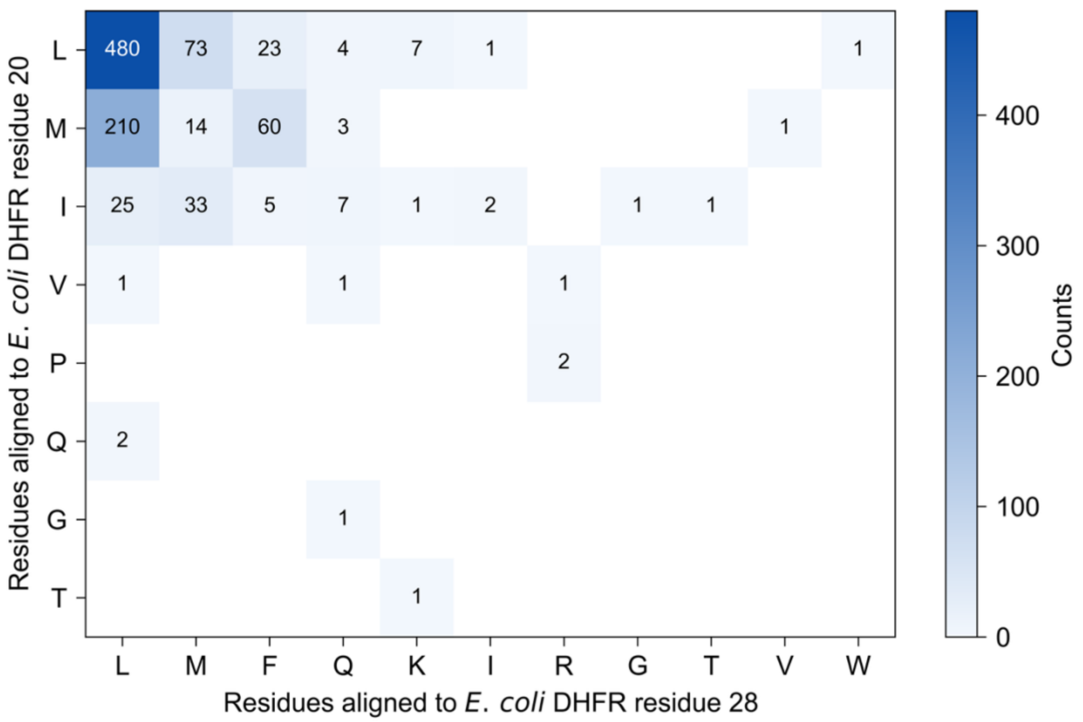

Supplementary Fig. 26. Distribution of homolog residue pairs aligned to *E. coli* DHFR positions 20 and 28. DHFR homologs from the lib15/Codon 1 library were categorized according to their amino acids at reference positions 20 and 28 (aligned to *E. coli* DHFR, UniProt: P0ABQ4). The heatmap displays the absolute count of homologs for each observed residue pair.

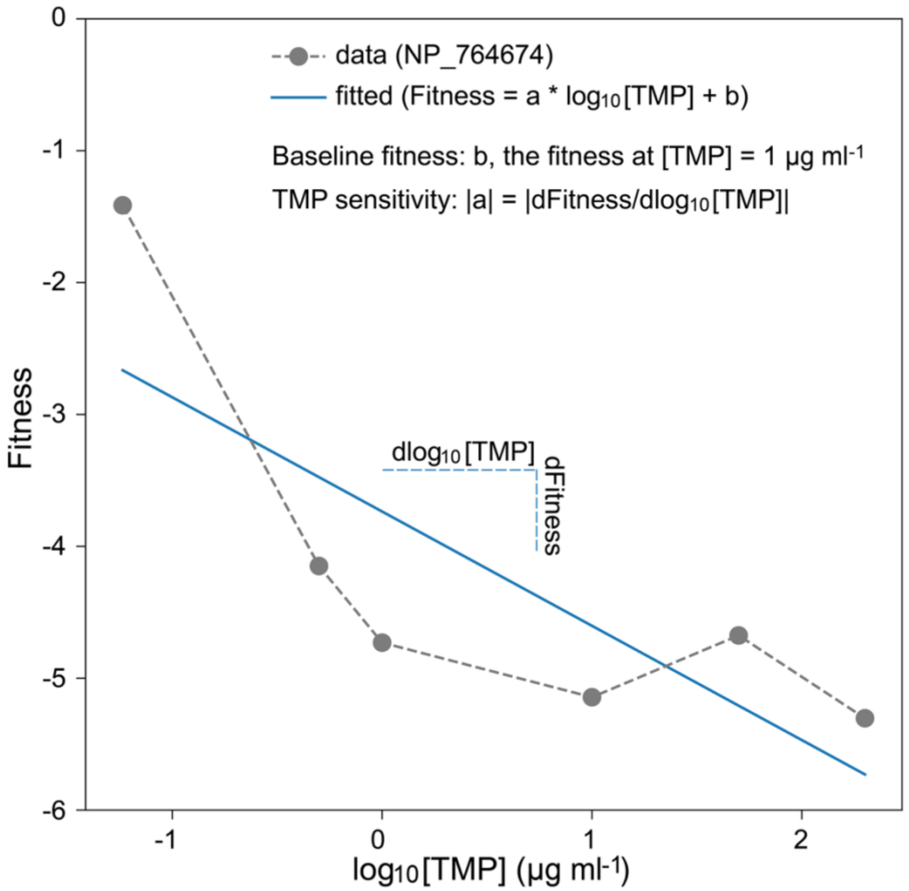

Supplementary Fig. 27. Schematic representation of TMP-response parameters derived from homolog fitness analyses. Grey points connected by a dashed line show the experimental fitness values for a representative homolog (NP_764674) across varying TMP concentrations. The solid blue line indicates the linear fitting. The intercept b was used as the fitted baseline fitness, corresponding to the estimated fitness at 1 μg ml^{-1} TMP. The absolute value of slope, $|a|$, was used as the TMP sensitivity, corresponding to the magnitude of fitness change per unit increase in $\log_{10}$[TMP].

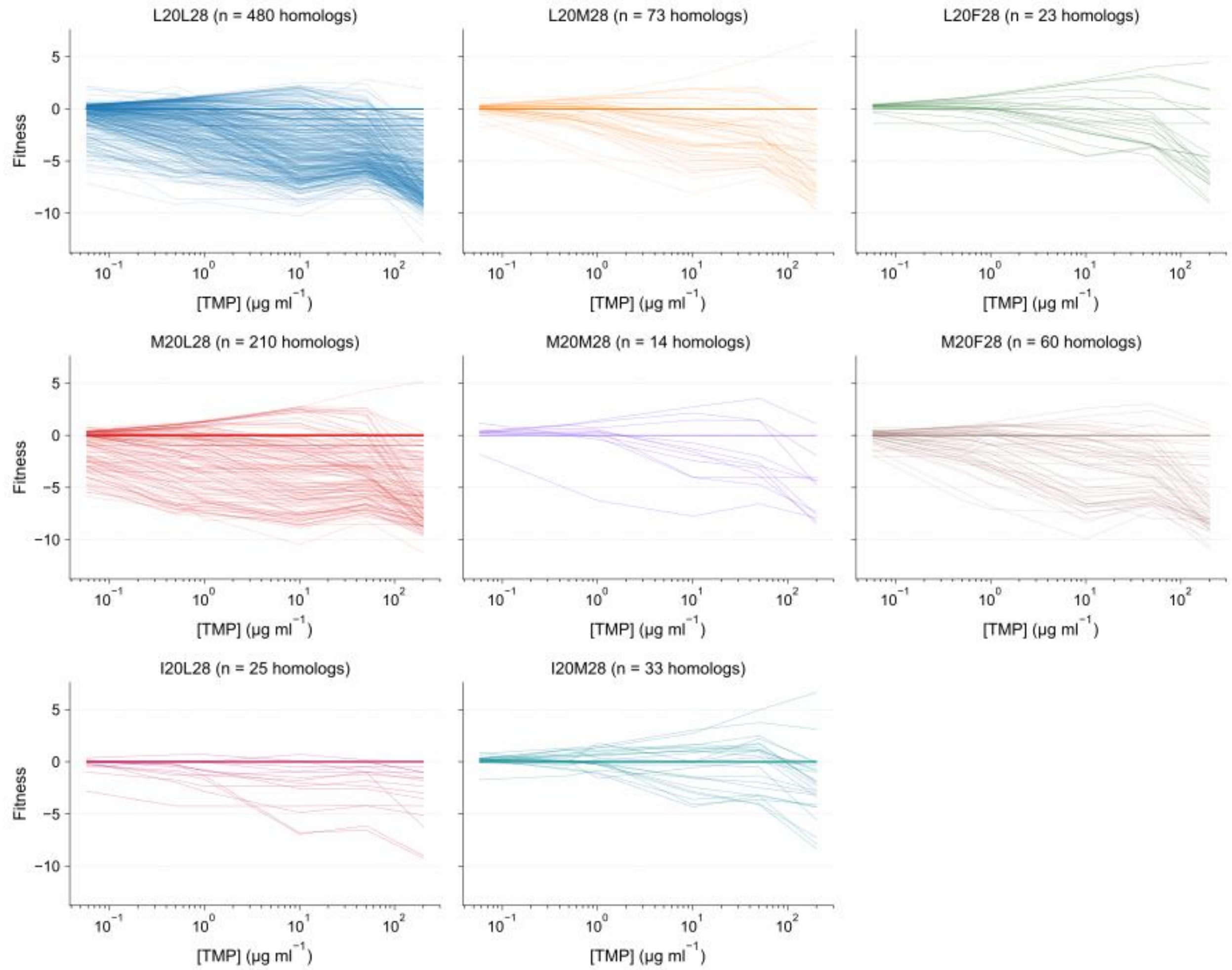

Supplementary Fig. 28. TMP-response curves of DHFR homologs grouped by position 20 and 28 residue pairs. Each line depicts the fitness trajectory of an individual homolog across varying TMP concentrations. Only residue-pair groups containing a minimum of 10 homologs are displayed.

Supplementary Table 1. Summary of DHFR promoter and coding sequence (CDS) mutations identified via directed evolution.

Notes: Trajectories 1–4 spanned approximately 60 days across 80 passages, whereas trajectories 5–16 spanned approximately 45 days across 60 passages. All trajectories were Sanger-sequenced at passage 60. For trajectories 1–4, an additional sequencing was performed at passage 80; mutations unique to this later time point, if any, are explicitly annotated.

	**GroEL/S Overexpression**		**Control**	
**Trajectory index**	**Promoter**	**CDS**	**Promoter**	**CDS**
**1**	none	W30R	none	none
**2**	T-15C	M20I @ Passage 60; M20I, L24R @ Passage 80	T-29C	none
**3**	none	P21L @ Passage 60; I5F @ Passage 80	none	none
**4**	T-35C	none	none	none
**5**	none	D27E, L28R	none	P21Q
**6**	none	none	none	L28R
**7**	none	P21L	none	none
**8**	none	none	none	D27E
**9**	T-5C	none	none	none
**10**	T-5C	P21L	T-29C	D27E
**11**	T-2C	none	none	A26T, D27E
**12**	T-5C	D27E	none	none
**13**	T-35C	none	A-9G	W30G, I94L
**14**	none	P21L	none	none
**15**	none	none	T-5C	D27E, F153C
**16**	A-4G	none	none	none

Supplementary Table 2. Kinetic model parameters.

Notation	Definition	Value in M20I model	Value in WT model	Comment
$[\mathbf{DHFR}]_A$	Concentration of active DHFR	NA	NA	
$[\mathbf{DHFR}]_I$	Concentration of inhibited DHFR	NA	NA	
$[\mathbf{DHFR}]_I^c$	Concentration of inhibited DHFR in closed state	NA	NA	
$[\mathbf{DHFR}]_I^o$	Concentration of inhibited DHFR in open state	NA	NA	
$[\mathbf{GroEL_{14}}:\mathbf{DHFR}$	Concentration of $GroEL_{14}$-DHFR complex	NA	NA	
$[\mathbf{GroEL_{14}}]$	Concentration of $GroEL_{14}$	2.2 μM	NA	1
$[\mathbf{DHFR}]_{total}$	Total concentration of DHFR in cytoplasm	0.4 μM	0.4 μM	2
$[\mathbf{TMP}]$	Concentration of TMP	88.57 μM	88.57 μM	3
k_{disso}	Dissociation rate constant of TMP separating from DHFR	0.058 s^{-1}	0.092 s^{-1}	4
k_{asso}	Association rate constant of TMP binding to DHFR	20 $\mu M^{-1} s^{-1}$	20 $\mu M^{-1} s^{-1}$	4
k_G	Reaction rate constant of GroEL/S refolding process	1/8 s^{-1}	NA	5
k_{on}	Association rate constant of $GroEL_{14}$ and DHFR	3.18×10^9 $M^{-1} s^{-1}$	NA	6
k_{off}	Dissociation rate constant of $GroEL_{14}$ and DHFR	Varied with K_d	NA	
ρ^c	Population of inhibited DHFR in closed state	NA	NA	
ρ^o	Population of inhibited DHFR in open state	NA	NA	
k_{co}	Closed-to-open rate constant	Varied	NA	
k_{oc}	Open-to-closed rate constant	Varied	NA	
K_d	Binding affinity of $GroEL_{14}$ and DHFR	Varied from 0.01 μM to 10 μM	NA	
$\Delta G_{closed-open}$	Free energy difference between closed-state and open-state	Varied with k_{co} and k_{oc}	NA	
k_{cat}	Catalytic constant of DHFR enzymatic reaction	6.8 s^{-1}	5.3 s^{-1}	7
K_M	Michaelis constant of DHFR enzymatic reaction	1.9 μM	2.2 μM	7
$[\mathbf{THF}]$	Concentration of tetrahydrofolate (THF)	NA	NA	

$[\mathbf{DHF}]$	Concentration of dihydrofolate (DHF)	10 μM	10 μM	8
$\boldsymbol{\eta}$	Viscosity of *E. coli* cytoplasm	3 mPa s	3 mPa s	9
$\boldsymbol{r_{GroEL_{14}}}$	Radius of gyration of GroEL14	6.6 nm	NA	10
$\boldsymbol{r_{DHFR}}$	Radius of gyration of DHFR	2 nm	NA	11
$\boldsymbol{T}$	Temperature	310 K	310 K	

Comments:

1. GroEL protomer abundance is obtained from PaxDb: Protein Abundance Database v6.0, *E. coli* str. K-12 substr. MG1655 - Whole organism (Integrated) dataset, with value as 6886 parts per million (ppm). Given *E. coli* contains 2.36x10^6 proteins per cell[1], there are around 16,251 GroEL protomers in each cell. Since *E. coli* cell volume is 0.86 μm^3 [1], *in vivo* GroEL protomer concentration is around 31.4 μM (i.e., $GroEL_{14}$ ~ 2.2 μM).
2. *In vivo* DHFR concentration is calculated in the same way as that of GroEL. DHFR abundance from PaxDb is 88.5 ppm, which converts to cellular concentration as 0.4 μM.
3. *In vivo* TMP concentration is obtained from Manna *et al.* (2021)[2], where *E. coli* BW25113 is treated with 5 μM TMP and harvested after 1 hour incubation. TMP concentration of 1 μM was used for numerical calculation in fig. 5e to mimic the condition used in fig. 3g.
4. Association rate constant between TMP and DHFR is obtained from Cayley *et al.* (1981)[3] with and dissociation rate constant calculated to reproduce measured K_i in fig. 2b.
5. Previous work places the ATP-driven GroEL/GroES reaction cycle on the timescale of several seconds, often approximated as ~8 s [4]; accordingly, we modeled the productive GroEL/S step with reaction rate constant as 1/8 s^{-1}.
6. The binding between $GroEL_{14}$ and its substrate proteins is close to diffusion-limited[5–7], therefore, we use diffusion rate constant k_{diff} as a proxy for the association rate constant of $GroEL_{14}$ and DHFR. Based on Smoluchowski equation,

$$k_{on} = k_{diff} = 4\pi\left(D_{GroEL_{14}} + D_{DHFR}\right)\left(r_{GroEL_{14}} + r_{DHFR}\right)$$

where

$$D = \frac{k_B T}{6\pi\eta r}$$

7. Measured by this work.
8. Since a canonical basal intracellular DHF concentration in *E. coli* is not well established, DHF was assumed to be 10 μM as an order-of-magnitude estimate for the modeling analysis. As DHF concentration was treated as a constant and the same value was used in both the WT and M20I models, this assumption does not affect the relative comparison between the two conditions.
9. The viscosity of the E. coli cytoplasm was based on a reported cytoplasmic microviscosity measured in live *E. coli* cells[8].
10. A radius of gyration of 2 nm was used for DHFR, consistent with the size expected for a compact folded protein of approximately 18 kDa and its structure.
11. A radius of gyration of 6.6 nm was used for $GroEL_{14}$, as reported by a solution X-ray scattering measurement for native *E. coli* $GroEL_{14}$[9].

Supplementary Table 3. Experimentally characterized GroEL-recognizable peptide motifs.

Notes: * Peptide designations match the original literature, defaulting to parent protein names where unspecified. ** In the absence of explicit K_d values, relative affinity comparisons are restricted to intra-study data. *** PAxpS90T serves as the reference sequence. Amino acid identities are represented by hyphens (–), while divergent positions are indicated by the single-letter code of the substituted residue.

Reference	Peptide*	Sequence	Secondary Structure	Binding strength**
10	N-terminal α-helix of rhodanese	STKWLAESVRAGK	α-helix	10-1000 μM (estimated)
11	vsv-C	KLIGVLSSLFRPK	α-helix	n/a
12	Residues 19–35 from eosinophil cationic protein (ECP)	NPPRCTIAMRAINNYRWR	n/a	n/a
13***	PAxpS90T (pS residues 67-86)	PLSTEALLKQVDYLIRSKWV	n/a	53 nM
	Gly5	-------GG-G-GG------		370 nM
	Gly4	-------GG-GG--------		100 nM
	Gly3	-------GG-G---------		36 nM
	Ser5	-------SS-S-SS------		196 nM
	Gln4-1	----------Q-QQQ-----		136 nM
	Gln4-2	-----QQQ--Q---------		96 nM
	Gln5	-----QQQ--Q----Q----		180 nM
	Pro2	---------P-P--------		37 nM
	Pro3-1	------P--P-P--------		62 nM
	Pro3-2	------P--P-----P----		72 nM
	Pro4	------P--P-P---P----		109 nM
	HCH 2	------R---D--RR----D		50 nM
	HCH 3	------RR------R-----		27 nM
	HCH 4	------RR------------		62 nM

	HCH 5	------RR--D--R------		62 nM
14	P23 (the N-terminal 23 amino acid signal sequence of pre-b-lactamase)	LRIQHFRVALIPFFAAFSLPVFG	n/a	10-100 nM
15	Rhodanese peptides	Two long peptides at about 7-9,000 Da and 11,000 Da	n/a	n/a
16	Gp31	AQAGDEEVTESGLIIGKRVQ	β-hairpin	n/a
17	APA08c (hydrophilic)	CNCK(FITC)APETKWCAESCRAGK	α-helix: 55%; β-strand: 45%	no binding detetcetd
	APA09r (hydrophobic)	CNCK(FITC)APETALCWLACSVRAGK	α-helix: 19%; β-strand: 81%	no binding detetcetd
	APA09c (hydrophobic)	CNCK(FITC)APETALCWLACSVRAGK	α-helix: 71%; β-strand: 29%	106 μM
	APA14c (hydrophobic)	CNCKAPETALCWLACSVRAGK	α-helix: 57%; β-strand: 27%; others: 16%	bound
18	Bamph (basic amphiphilic)	PLYKKIIKKLLES	n/a	0.54 μM
	Namph (neutral amphiphilic)	PLYQQIIQQLLNS		3.57 μM
	Aamph (acidic amphiphilic)	PLYEEIIEELLES		>10 μM
	Ahphil (acidic hydrophilic)	PQYEENNEEQQES		>10 μM
	Bhphil (basic hydrophilic)	PQYKKNNKKQQES		3.44 μM
19	7 residues of a 17-residue N-terminal tag attached to the mini GroEL	GLVPRGS	Extended conformation	n/a
20	GroES	KRKEVETK AGGIVLTGSAA	β-hairpin	n/a
21	AMPH+	ALYKKIIKKLLESK	α-helix	5 nM
	AMPH-	ALYKKIIKKLLESK	less α-helix	51 nM

	AMPHR	ALYKKIIKKLLESK	unstructured	196 nM
	NON-AMPH+	ALYKIKKIKLLESK	α-helix	62 nM
	NON-AMPH-	ALYKIKKIKLLESK	unstructured	83 nM
	NON-AMPHR	ALYKIKKIKLLESK	unstructured	215 nM
22	P22 tailspike	DYVKFPGIETLL	n/a	+++
	vsv-C	KLIGVLSSLFRPK	α-helix	+++
	Rho2-L6	STKWLLESVRAGK	n/a	+++
	AL	YKALSEALKSAK	n/a	+++
	ID (amphipathic variants of Rho2)	YKALAESLKSAK	α-helix	+++
	Rho2	STKWLAESVRAGK	α-helix	+++
	D-Rho2 (chirality variants of Rho2)	STKWLAESVRAGK	Left-handed α-helix	+++
	Xhat1	YSTATLSLGHHAVP	β-strand	+++
	Rho2-5P6	STKWLPAESVRAGK	n/a	+++
	L,D-Rho2 (chirality variants of Rho2)	STKWLAESVRAGK	unable to form α-helix	+++
	KE (non-amphipathic variants of Rho2)	YKLSAEKLSAAK	α-helix	++
	ES24D	KRKEVETKSAGDIVLTGSAA	n/a	++
	ES24G	KRKEVETKSAGGIVLTGSAA	n/a	++
	Rho2-P5	STKWPAESVRAGK	n/a	++
	"control"	GPENRGDSCA	n/a	++
	Rho2-P5G6	STKWPGESVRAGK	n/a	++
	CRAPB3	SKPHVEIRQDGD	β-strand	+
	KWK	KWK	n/a	+
23	Rho (N-terminal α-helix of rhodanese)	STKWLAESVRAGK	α-helix	n/a

	SBP (strongly binding peptide)	FHYEIWIPPHRG	β-hairpin	2 μM
24	1	SWMTTPWGFLHP	n/a	n/a
	3	SSPWWLVSFTST		
	4	SHSLIWRIPLLH		
	5	IYVPWYYAENLP		
	6	YNYSWNGVVFVP		
	7	AQSTPLMKPQKS		
	8	DQTTLQRFLGSH		
	9	QTIKPPITVHPS		
	10	QYNHILGYLPFQ		
	11	IMDPQNSKVTVA		
	12	LPIQNAKRSMVS		
	13	IMSPWDESFWNY		
	14	ASESYVLFPGTR		
	15	SNWHGPLSYQLM		
	16	ALPLQDTAATLS		
	17	QEIYLTPRGPQQ		
	18	IDRTQMWRQSDL		
	19	INRDHPLHAGQP		
	20	HQTPQSLARWSL		
	21	HSLRAIQLITGM		
25	Peptide 1 in Ref. 24	SWMTTPWGFLHP	β-hairpin	n/a
26	SBP-W2DP6V	SDMTTVWGFLHP	310/α-helix	17.1 μM
27	Peptide 1 in Ref. 24	SWMTTPWGFHLP	β-hairpin	n/a

Supplementary Table 4. X-ray crystallography statistics, related to Fig. 4a.

	M20I:NADPH:TMP
PDB ID	XXXX
Wavelength (Å)	1.541840
Resolution range	49.53 - 1.898 (1.966 - 1.898)
Space group	P 21 21 21
Unit cell	34.1561 45.5504 99.0501 90 90 90
Total reflections	86516 (6206)
Unique reflections	12754 (1034)
Multiplicity	6.8 (5.2)
Completeness (%)	98.47 (85.31)
Mean I/sigma(I)	4.81 (0.36)
Wilson B-factor	20.39
R-merge	0.1756 (0.7688)
R-meas	0.19 (0.8551)
R-pim	0.07142 (0.3658)
CC1/2	0.992 (0.686)
CC*	0.998 (0.902)
Reflections used in refinement	12580 (1034)
Reflections used for R-free	653 (51)
R-work	0.1885 (0.2815)
R-free	0.2343 (0.3403)
CC (work)	0.960 (0.797)
CC (free)	0.947 (0.597)
Number of non-hydrogen atoms	1520
Macromolecules	1353
Ligands	119
Solvent	92
Protein residues	159
Nucleic acid bases	
RMS (bonds)	0.021
RMS (angles)	1.74
Ramachandran favored (%)	97.45
Ramachandran allowed (%)	2.55
Ramachandran outliers (%)	0
Rotamer outliers (%)	2.07
Clashscore	8.96
Average B-factor	21.79
Macromolecules	21.55
Ligands	18.84
Solvent	27.76

Supplementary Table 5. Reagent and Resources.

Reagent of Resource	Source	Identifier
Terrific Broth (TB)	BD Difco™	243820
LB broth	Millipore®	1.10285
M9 minimal salts	BD Difco™	248510
Glycerol	VWR Chemicals BDH®	BDH1172
D-(+)-Glucose monohydrate	Millipore®	49159
MOPS	Sigma-Aldrich®	M3183
Citric acid	VWR Chemicals BDH®	BDH4136
Ferric ammonium citrate	VWR AMRESCO®	0846
Magnesium sulfate ($MgSO_4$)	Sigma-Aldrich®	M2643
Calcium chloride ($CaCl_2$)	VWR Chemicals BDH®	BDH4122
Arabinose	Alfa Aesar	A11921
Trimethoprim	Sigma-Aldrich®	T7883
isopropyl β-D-1-thiogalactopyranoside (IPTG)	RPI	I56000
Kanamycin sulfate	Millipore®	420311
Streptomycin sulfate salt	Sigma-Aldrich®	S9137
Ampicillin	VWR	0339
Chloramphenicol	Calbiochem®	220551
Anhydrotetracycline (aTc)	Takara	631310
Bicine	Sigma-Aldrich®	B3876
Imidazole	Millipore®	5710-OP
Sodium chloride (NaCl)	VWR Chemicals BDH®	BDH9286
Potassium chloride (KCl)	Sigma-Aldrich®	P3911
Tris base	Sigma-Aldrich®	T4661
Tris-Buffered Saline (TBS)	Corning®	46-012-CM
Phosphate Buffered Saline (PBS)	Corning®	46-013-CM
Benzonase® Nuclease	Millipore®	70664
cOmplete™, Mini, EDTA-free Protease Inhibitor Cocktail	Roche	11836170001
Lysozyme	Thermo Scientific™	89833
TCEP Hydrochloride	RPI	T26500
0.5 M EDTA	Invitrogen™	AM9261
Dithiothreitol (DTT)	RPI	D11000
cOmplete™ His-Tag Purification Resin	Roche	5893682001
Methotrexate (MTX)-Agarose	Sigma-Aldrich®	M0269
Ammonium sulfate ($(NH_4)_2SO_4$)	Sigma-Aldrich®	09978
Monopotassium phosphate (KH_2PO_4)	G-Biosciences	RC084
Dipotassium phosphate (K_2HPO_4)	G-Biosciences	RC081
Potassium tetraborate tetrahydrate ($K_2B_4O_7 \cdot 4H_2O$)	Sigma-Aldrich®	P5754
HEPES sodium salt	Sigma-Aldrich®	H3784

D-Glucose (U-$^{13}C_6$)	Cambridge Isotope Laboratories	CLM-1396
Ammonium chloride (^{15}N)	Cambridge Isotope Laboratories	NLM-467
Ammonium sulfate ($^{15}N_2$)	Cambridge Isotope Laboratories	NLM-713
Deuterium oxide "100%" (D, 99.96%) +0.01 mg/mL DSS	Cambridge Isotope Laboratories	DLM-6DB-10X0.7
MEM Vitamins 100x Solution	Corning®	25-020-CI
Acetone	VWR Chemicals BDH®	BDH1101
L-Malate Dehydrogenase (L-MDH)	Roche	10127914001
Pyruvate Kinase (PK)	Roche	10128155001
Phosphoenol-pyruvate (PEP-K)	Roche	10108294001
NADP	Roche	10128031001
Oxaloacetic acid	Sigma-Aldrich®	O7753
Magnesium acetate	VWR Chemicals BDH®	BDH4170
bis-ANS	Invitrogen™	B153
NADPH	Roche	10621706001
Dihydrofolic acid (DHF)	Sigma-Aldrich®	D7006
Sodium hydroxide (NaOH)	Macron	7708-10
Dimethyl sulfoxide (DMSO)	Sigma-Aldrich®	276855
Adenosine 5′-triphosphate disodium salt hydrate (ATP)	Sigma-Aldrich®	A2383
EveryBlot Blocking Buffer	Bio-Rad	12010020
Urea	Sigma-Aldrich®	RES9692U-A102X
Guanidine hydrochloride (GdnHCl)	Sigma-Aldrich®	G3272
DSP	Thermo Scientific™	A35393
B-PER™ II Bacterial Protein Extraction Reagent	Thermo Scientific™	78260
Tween-20	Sigma-Aldrich®	P9416
Thiamine hydrochloride	Sigma-Aldrich®	T1270
Casamino acids	Teknova	C2000
D-Glucose 6-phosphate sodium salt	Sigma-Aldrich®	G7879
Glucose-6-phosphate Dehydrogenase	Sigma-Aldrich®	G6378
Rabbit anti-DHFR antibody	Pacific Immunology	Customized
HRP-linked Goat anti-Rabbit IgG	Bio-Rad	STAR124P
HRP-linked Horse Anti-Mouse IgG	Cell Signaling Technology	7076S
Mouse anti-GroEL antibody	abcam	ab82592
Rabbit anti-GroEL antibody	abcam	ab90522
Pierce™ Protein A/G Magnetic Beads	Thermo Scientific™	88803
HiLoad 16/600 Superdex 75 pg column	Cytiva	28989333
HiPrep Q Fast Flow 16/10 column	Cytiva	28936543
HiLoad 16/600 Superose 6 pg column	Cytiva	29323952

Superdex 200 Increase 10/300 GL column	Cytiva	28990944
XK 16/20 Column	Cytiva	28988937
Amicon® Stirred Cell	Millipore®	UFSC40001
Low Pressure/Vacuum (LPV) NMR tube	SP Wilmad®	535-LPV-200M

Supplementary Table 6. Plasmids.

Plasmid	Vector	Protein encoded	Comments
pET-28a(+)-DHFR-6His	pET-28a(+)	*E. coli* DHFR (WT and single mutants)	Synthesized by GenScript Biotech (Nanjing, China); for protein purification.
pET-28a(+)-DHFR	pET-28a(+)	*E. coli* DHFR (WT and M20I)	Synthesized by GenScript Biotech (Nanjing, China) with C-terminal 6×His-tag removed; for protein purification.
pOA-GroEL/S	pOA	*E. coli* GroEL (no tag) and *E. coli* GroES (C-terminal 6×His-tag)	Gift from Dr. Shimon Bershtein; for protein purification.
pGro7	N/A	*E. coli* GroEL and *E. coli* GroES	Obtained from Takara Bio (Cat. #3340); for GroEL/S overexpression.
pEmpty	N/A	None	Constructed from pGro7; control.
pdCas9-bacteria	p15A	Catalytically inactive bacterial Cas9 (S. pyogenes)	pdCas9-bacteria was a gift from Stanley Qi[28] (Addgene plasmid # 44249 ; http://n2t.net/addgene:44249 ; RRID:Addgene_44249).
pgRNA-bacteria-groSP	pUC19	N/A	pgRNA-bacteria was a gift from Stanley Qi[28] (Addgene plasmid # 44251 ; http://n2t.net/addgene:44251 ; RRID:Addgene_44251).<br><br>Customized guide RNA (gRNA): tgaccagagaaatggggatg (5'→3').
pgRNA-bacteria-empty	pUC19	N/A	pgRNA-bacteria was a gift from Stanley Qi[28] (Addgene plasmid # 44251 ; http://n2t.net/addgene:44251 ; RRID:Addgene_44251).<br><br>Guide RNA (gRNA) was removed.
pfolA-GFP	N/A	Green fluorescent protein (GFP)	Obtained from Horizon Discovery (Cat. #PEC3876-202384323)

Supplementary Table 7. Primers.

Primer	Sequence (5'→3')	Comments
pEmpty_fwd	taattgccctgcacctcg	Construct pEmpty from pGro7
pEmpty_rvs	tgataactctcctttgagaaagtccg	
pred_groSp_fwd	AATGGGGATGgttttagagctagaaatagcaag	Construct pgRNA-bacteria-groSP from pgRNA-bacteria
pred_groSp_rev	TCTCTGGTCAactagtattatacctaggactg	
empty_gRNA_fwd	gttttagagctagaaatagcaag	Construct pgRNA-bacteria-empty from pgRNA-bacteria
empty_gRNA_rev	actagtattatacctaggactg	
pfolA_c-2t_GFP_FWD	AAAAAAAATTaTCGCCACTATACG	Introduce c-2t mutation to folA promoter
pfolA_c-2t_GFP_REV	ATCGGGAAATCTCAATG	
pfolA_g-4a_GFP_FWD	AAAAAATTGTtGCCACTATACG	Introduce g-4a mutation to folA promoter
pfolA_g-4a_GFP_REV	TTATCGGGAAATCTCAATG	
pfolA_c-5t_GFP_FWD	AAAAATTGTCaCCACTATACGTAAAG	Introduce c-5t mutation to folA promoter
pfolA_c-5t_GFP_REV	TTTATCGGGAAATCTCAATG	
pfolA_g-9a_GFP_FWD	ATTGTCGCCAtTATACGTAAAG	Introduce g-9a mutation to folA promoter
pfolA_g-9a_GFP_REV	TTTTTTTATCGGGAAATCTC	
pfolA_c-15t_GFP_FWD	GCCACTATACaTAAAGCGTAAAC	Introduce c-15t mutation to folA promoter
pfolA_c-15t_GFP_REV	GACAATTTTTTTATCGGGAAATC	
pfolA_c-29t_GFP_FWD	AGCGTAAACCaTCGTCGACTG	Introduce c-29t mutation to folA promoter
pfolA_c-29t_GFP_rev	TTACGTATAGTGGCGAC	
pfolA_c-35t_GFP_FWD	AACCGTCGTCaACTGGTGCGA	Introduce c-35t mutation to folA promoter
pfolA_c-35t_GFP_REV	TACGCTTTACGTATAGTGGC	

Supplementary Table 8. Bacterial strains.

Strain	Genotype	Plasmid	Comments
E. coli BL21(DE3)	/	None	NEB (Cat. #C2527)
E. coli DH5α	/	None	NEB (Cat. #C2987)
E. coli BW25113	/	None	CGSC: 7636
E. coli TG1/pOA	*E. coli* TG1	pOA-GroEL/S	Gift from Dr. Shimon Bershtein
JL01	BW25113 *folA*(M20I)	None	This study
JL02	BW25113 *folA*(L28R)	None	This study
JL03	BW25113 *folA*(W30C)	None	This study
JL04	BW25113	pdCas9-bacteria; pgRNA-bacteria-groSP	This study
JL05	BW25113	pdCas9-bacteria; pgRNA-bacteria-empty	This study
JL06	BW25113 *folA*(M20I)	pdCas9-bacteria; pgRNA-bacteria-groSP	This study
JL07	BW25113 *folA*(M20I)	pdCas9-bacteria; pgRNA-bacteria-empty	This study
JL08	BW25113 *folA*(L28R)	pdCas9-bacteria; pgRNA-bacteria-groSP	This study
JL09	BW25113 *folA*(L28R)	pdCas9-bacteria; pgRNA-bacteria-empty	This study
JL10	BW25113 *folA*(W30C)	pdCas9-bacteria; pgRNA-bacteria-groSP	This study
JL11	BW25113 *folA*(W30C)	pdCas9-bacteria; pgRNA-bacteria-empty	This study
JL12	BW25113	pGro7	This study

JL13	BW25113	pEmpty	This study
JL14	BW25113 *folA*(M20I)	pGro7	This study
JL15	BW25113 *folA*(M20I)	pEmpty	This study

Reference

1. Milo, R. What is the total number of protein molecules per cell volume? A call to rethink some published values. *BioEssays* **35**, 1050–1055 (2013).
2. Manna, M. S. *et al.* A trimethoprim derivative impedes antibiotic resistance evolution. *Nat. Commun.* **12**, 2949 (2021).
3. Cayley, P. J., Dunn, S. M. J. & King, R. W. Kinetics of substrate, coenzyme, and inhibitor binding to Escherichia coli dihydrofolate reductase. *Biochemistry* **20**, 874–879 (1981).
4. Ueno, T., Taguchi, H., Tadakuma, H., Yoshida, M. & Funatsu, T. GroEL Mediates Protein Folding with a Two Successive Timer Mechanism. *Mol. Cell* **14**, 423–434 (2004).
5. Perrett, S., Zahn, R., Stenberg, G. & Fersht, A. R. Importance of electrostatic interactions in the rapid binding of polypeptides to GroEL1. *J. Mol. Biol.* **269**, 892–901 (1997).
6. Sparrer, H., Lilie, H. & Buchner, J. Dynamics of the GroEL – Protein Complex: Effects of Nucleotides and Folding Mutants. *J. Mol. Biol.* **258**, 74–87 (1996).
7. Gray, T. E. & Fersht, A. R. Refolding of Barnase in the Presence of GroE. *J. Mol. Biol.* **232**, 1197–1207 (1993).
8. Chen, E., Esquerra, R. M., Meléndez, P. A., Chandrasekaran, S. S. & Kliger, D. S. Microviscosity in E. coli Cells from Time-Resolved Linear Dichroism Measurements. *J. Phys. Chem. B* **122**, 11381–11389 (2018).
9. Igarashi, Y. *et al.* Solution X-ray scattering study on the chaperonin GroEL from *Escherichia coli*. *Biophys. Chem.* **53**, 259–266 (1995).
10. Landry, S. J. & Gierasch, L. M. The chaperonin GroEL binds a polypeptide in an .alpha.-helical conformation. *Biochemistry* **30**, 7359–7362 (1991).
11. Landry, S. J., Jordan, R., McMacken, R. & Gierasch, L. M. Different conformations for the same polypeptide bound to chaperones DnaK and GroEL. *Nature* **355**, 455–457 (1992).

12. Rosenberg, H. F., Ackerman, S. J. & Tenen, D. G. Characterization of a distinct binding site for the prokaryotic chaperone, GroEL, on a human granulocyte ribonuclease. *J. Biol. Chem.* **268**, 4499–4503 (1993).

13. Dessauer, C. W. & Bartlett, S. G. Identification of a chaperonin binding site in a chloroplast precursor protein. *J. Biol. Chem.* **269**, 19766–19776 (1994).

14. Zahn, R. *et al.* Thermodynamic Partitioning Model for Hydrophobic Binding of Polypeptides by GroEL: I. GroEL Recognizes the Signal Sequences of β-lactamase Precursor. *J. Mol. Biol.* **242**, 150–184 (1994).

15. Hlodan, R., Tempst, P. & Hartl, F. U. Binding of defined regions of a polypeptide to GroEL and its implications for chaperonin-mediated protein folding. *Nat. Struct. Biol.* **2**, 587–595 (1995).

16. Landry, S. J., Taher, A., Georgopoulos, C. & van der Vies, S. M. Interplay of structure and disorder in cochaperonin mobile loops. *Proc. Natl. Acad. Sci.* **93**, 11622–11627 (1996).

17. Brazil, B. T. *et al.* Model Peptide Studies Demonstrate That Amphipathic Secondary Structures Can Be Recognized by the Chaperonin GroEL (cpn60)*. *J. Biol. Chem.* **272**, 5105–5111 (1997).

18. Hutchinson, J. P., Oldham, T. C., El-Thaher, T. S. H. & Miller, A. D. Electrostatic as well as hydrophobic interactions are important for the association of Cpn60 (groEL) with peptides. *J. Chem. Soc. Perkin Trans. 2* 279–288 (1997) doi:10.1039/a604880c.

19. Buckle, A. M., Zahn, R. & Fersht, A. R. A structural model for GroEL–polypeptide recognition. *Proc. Natl. Acad. Sci.* **94**, 3571–3575 (1997).

20. Xu, Z., Horwich, A. L. & Sigler, P. B. The crystal structure of the asymmetric GroEL–GroES–(ADP)7 chaperonin complex. *Nature* **388**, 741–750 (1997).

21. Preuss, M., Hutchinson, J. P. & Miller, A. D. Secondary Structure Forming Propensity Coupled with Amphiphilicity Is an Optimal Motif in a Peptide or Protein for Association with Chaperonin 60 (GroEL). *Biochemistry* **38**, 10272–10286 (1999).

22. Wang, Z., Feng, H., Landry, S. J., Maxwell, J. & Gierasch, L. M. Basis of Substrate Binding by the Chaperonin GroEL. *Biochemistry* **38**, 12537–12546 (1999).

23. Kobayashi, N., Freund, S. M. V., Chatellier, J., Zahn, R. & Fersht, A. R. NMR analysis of the binding of a rhodanese peptide to a minichaperone in solution1. *J. Mol. Biol.* **292**, 181–190 (1999).

24. Chen, L. & Sigler, P. B. The Crystal Structure of a GroEL/Peptide Complex: Plasticity as a Basis for Substrate Diversity. *Cell* **99**, 757–768 (1999).

25. Wang, J. & Chen, L. Domain Motions in GroEL upon Binding of an Oligopeptide. *J. Mol. Biol.* **334**, 489–499 (2003).

26. Li, Y., Gao, X. & Chen, L. GroEL Recognizes an Amphipathic Helix and Binds to the Hydrophobic Side*. *J. Biol. Chem.* **284**, 4324–4331 (2009).

27. Izert-Nowakowska, M. A. *et al.* Targeted protein degradation in Escherichia coli using CLIPPERs. *EMBO Rep.* **26**, 3994–4016 (2025).

28. Qi, L. S. *et al.* Repurposing CRISPR as an RNA-Guided Platform for Sequence-Specific Control of Gene Expression. *Cell* **152**, 1173–1183 (2013).
